# Supplementary material for: Can Siberian alder N-fixation offset N-loss after severe fire? Quantifying post-fire Siberian alder distribution, growth, and N-fixation in boreal Alaska
Source: PLoS One. 2020 Sep 2;15(9):e0238004. doi: 10.1371/journal.pone.0238004 (PMC7467271; doi:10.1371/journal.pone.0238004)
Supplement: S1 Table — Statistics were calculated using the 2015 dataset (n = 21). Variables significantly different (p < 0.05) across stand types are shown in bold print. Different letters in the same row indicate significant differences among stand types at p < 0.05. NODBIO = live nodule biomass (g nodule m-2 plant-1); Height = mean ramet height (m); SLM = specific leaf mass (mg cm-2); MRD = mean ramet diameter (cm); LRPP = live ramets per plant; DRPP = dead ramets per plant; PCA1 = PCA axis 1 [plant-level live nodule biomass (+), mean ramet height (+), mean ramet diameter (+), and specific leaf mass (-)]; PCA2 = PCA axis 2 [number of live ramets per plant (+) and dead ramets per plant (+)]. Values reflect mean ± standard error. (DOCX) [file pone.0238004.s001.docx]

| Variable | Black Spruce  (n = 8) | Deciduous  (n = 5) | Mixed  (n = 8) | ANOVA |
| --- | --- | --- | --- | --- |
| NODBIO | 6.3 ± 2.0 a | 13.4± 3.2 a | 10.2 ± 2.8 a | F(2,18) = 1.622, p = 0.225 |
| **Height** | **1.5 ± 0.1 a** | **3.7 ± 0.3 b** | **2.2 ± 0.4 a** | **F(2,18) = 8.277, p = 0.003** |
| **SLM** | **7.6 ± 0.2 a** | **5.0 ± 0.3 b** | **6.9 ± 0.5 a** | **F(2,18) = 10.362, p = 0.001** |
| **MRD** | **1.8 ± 0.1 a** | **4.4 ± 0.4 b** | **2.5 ± 0.2 c** | **F(2,18) = 22.870, p = 0.00001** |
| LRPP | 11.9 ± 2.0 a | 16.2 ± 2.9 a | 14.8 ± 4.9 a | F(2,18) =0.520, p = 0.603 |
| DRPP | 2.2 ± 0.7 a | 2.5 ± 0.6 a | 2.7 ± 1.2 a | F(2,18) = 0.198, p = 0.822 |
| **PCA1** | **-0.68 ± 0.09 a** | **1.42 ± 0.22 b** | **-0.06 ± 0.33 a** | **F(2,18) = 12.060, p = 0.0004** |
| PCA2 | 0.25 ± 0.31 a | 0.66 ± 0.26 a | 0.55 ± 0.55 a | F(2,18) = 0.454, p = 0.642 |
