# Supplementary material for: Can Siberian alder N-fixation offset N-loss after severe fire? Quantifying post-fire Siberian alder distribution, growth, and N-fixation in boreal Alaska
Source: PLoS One. 2020 Sep 2;15(9):e0238004. doi: 10.1371/journal.pone.0238004 (PMC7467271; doi:10.1371/journal.pone.0238004)
Supplement: S2 Table — The PCA was conducted with 2015 plots (n = 40) and includes: plant-level live nodule biomass (g nodule m-2 plant-1), mean ramet height (m), specific leaf mass (g cm-2), mean ramet diameter (cm), and a count of live and dead ramets per plant. Eigenvalue cutoff was set at 1. (DOCX) [file pone.0238004.s002.docx]

|  | Initial Eigenvalues | | | Extraction Sums of Squared Loadings | | | Rotation Sums of Squared Loadings | | |
| --- | --- | --- | --- | --- | --- | --- | --- | --- | --- |
| Axis | Total | % of Variance | Cumulative % | Total | % of Variance | Cumulative % | Total | % of Variance | Cumulative % |
| 1 | 2.895 | 48.257 | 48.257 | 2.895 | 48.257 | 48.257 | 2.761 | 46.017 | 46.017 |
| 2 | 1.534 | 25.561 | 73.818 | 1.534 | 25.561 | 73.818 | 1.668 | 27.802 | 73.818 |
| 3 | .707 | 11.777 | 85.595 |  |  |  |  |  |  |
| 4 | .397 | 6.622 | 92.217 |  |  |  |  |  |  |
| 5 | .305 | 5.083 | 97.300 |  |  |  |  |  |  |
| 6 | .162 | 2.700 | 100.000 |  |  |  |  |  |  |
