# Supplementary material for: Can Siberian alder N-fixation offset N-loss after severe fire? Quantifying post-fire Siberian alder distribution, growth, and N-fixation in boreal Alaska
Source: PLoS One. 2020 Sep 2;15(9):e0238004. doi: 10.1371/journal.pone.0238004 (PMC7467271; doi:10.1371/journal.pone.0238004)
Supplement: S3 Table — Statistics were calculated with the 2015 dataset (n = 40) unless stated otherwise (2014 dataset, n = 200). Significant differences between the Boundary Fire and Wickersham Dome Fire are determined at p < 0.05 level and shown in bold font. Statistics are calculated for plots in which alder was present. NODBIO = live nodule biomass (g nodule m-2 plant-1); SLM = specific leaf mass (mg cm-2); Height = mean ramet height (m); MRD = mean ramet diameter (cm); LRPP = live ramets per plant; DRPP = dead ramets per plant; PCA1 = PCA axis 1 [plant-level live nodule biomass (+), mean ramet height (+), mean ramet diameter (+), and specific leaf mass (-)]; PCA2 = PCA axis 2 [number of live ramets per plant (+) and dead ramets per plant (+)]. Values reflect mean ± standard error. (DOCX) [file pone.0238004.s003.docx]

| Variable | BF  (n = 19) | WDF  (n = 21) | ANOVA or Kruskal Wallis Test |
| --- | --- | --- | --- |
| NODBIO | 16.3 ± 3.6 | 9.5 ± 1.6 | F(1,38) = 0.777, p = 0.384 |
| SLM | 6.9 ± 0.3 | 6.7 ± 0.3 | F(1,38) = 0.244, p = 0.625 |
| **Height (n = 200)** | **1.7 ± 0.1** | **2.3 ± 0.1** | **χ^2^ (1, N = 200) = 15.471, p = 8.378e-05** |
| **MRD (n = 200)** | **2.0 ± 0.1** | **2.8 ± 0.1** | **χ^2^ (1, N = 200) = 38.933, p = 4.386e-10** |
| **LRPP (n = 200)** | **7.4 ± 0.4** | **14.3 ± 1.1** | **F(1,198) = 36.04, p = 9.09e-09** |
| **DRPP (n = 200)** | **0.6 ± 0.1** | **2.7 ± 0.3** | **χ^2^ (1, N = 200) = 67.356, p = 2.267e-16** |
| PCA1 | -0.06 ± 0.23 | 0.06 ± 0.12 | F(1,38) = 0.143, p = 0.708 |
| **PCA2** | **-0.51 ± 0.12** | **0.46 ± 0.24** | **χ^2^ (1, N = 40) = 10.651, p = 0.0011** |
