# Supplementary material for: Can Siberian alder N-fixation offset N-loss after severe fire? Quantifying post-fire Siberian alder distribution, growth, and N-fixation in boreal Alaska
Source: PLoS One. 2020 Sep 2;15(9):e0238004. doi: 10.1371/journal.pone.0238004 (PMC7467271; doi:10.1371/journal.pone.0238004)
Supplement: S4 Table — Statistics were calculated using the 2015 dataset (n = 19), unless stated otherwise (2014 dataset, n = 125). Variables significantly different (p < 0.05) across stand types are shown in bold print. Different letters among columns in the same row indicate significant differences among stand types at p < 0.05. NODBIO = live nodule biomass (g nodule m-2 plant-1); Height = mean ramet height (m); SLM = specific leaf mass (mg cm-2); MRD = mean ramet diameter (cm); LRPP = live ramets per plant; DRPP = dead ramets per plant; PCA1 = PCA axis 1 [plant-level live nodule biomass (+), mean ramet height (+), mean ramet diameter (+), and specific leaf mass (-)]; PCA2 = PCA axis 2 [number of live ramets per plant (+) and dead ramets per plant (+)]. Values reflect mean ± standard error. (DOCX) [file pone.0238004.s004.docx]

| Variable | Deciduous-Moderate  (n = 8) | Black Spruce-Moderate  (n = 3) | Black Spruce-  Moderate to High  (n = 5) | Black Spruce-High  (n = 3) | ANOVA |
| --- | --- | --- | --- | --- | --- |
| NODBIO | 23.0 ± 5.7 a | 4.1 ± 3.6 a | 16.6 ± 8.4 a | 10.1 ± 4.5 a | F(3,15) = 1.607, p = 0.230 |
| Height | 2.3 ± 0.3 a | 1.2 ± 0.1 a | 1.7 ± 0.2 a | 1.7 ± 0.1 a | F(3,15) = 2.288, p = 0.120 |
| **SLM** | **5.8 ± 0.2 a** | **8.3 ± 0.6 b** | **7.6 ± 0.5 b** | **7.5 ± 0.3 b** | **F(3,15) = 8.671, p = 0.001** |
| MRD | 2.8 ± 0.4 a | 1.3 ± 0.2 a | 1.5 ± 0.3 a | 1.7 ± 0.2 a | F(3,15) = 3.125, p = 0.0573 |
| **LRPP** | **12.2 ± 3.0 a** | **5.7 ± 0.7 ab** | **4.0 ± 0.6 b** | **3.4 ± 0.8 b** | **F(3,15) = 4.809, p = 0.0153** |
| DRPP | 0.8 ± 0.3 a | 0.7 ± 0.2 a | 0.6 ± 0.4 a | 1.3 ± 1.3 a | F(3,15) = 0.278, p = 0.84 |
| **PCA1** | **0.67 ± 0.33 a** | **-1.04 ± 0.21 b** | **-0.38 ± 0.38 ab** | **-0.50 ± 0.07 ab** | **F(3,15) = 4.421, p = 0.0204** |
| PCA2 | -0.32 ± 0.22 a | -0.52 ± 0.10 a | -0.81 ± 0.21 a | -0.53 ± 0.35 a | F(3,15) = 0.829, p = 0.498 |
