# Supplementary material for: Can Siberian alder N-fixation offset N-loss after severe fire? Quantifying post-fire Siberian alder distribution, growth, and N-fixation in boreal Alaska
Source: PLoS One. 2020 Sep 2;15(9):e0238004. doi: 10.1371/journal.pone.0238004 (PMC7467271; doi:10.1371/journal.pone.0238004)
Supplement: S1 File — (ZIP) [file pone.0238004.s005.zip › AIC_BF_density.docx]

> ## min_dist model in boundary fire

> bmin_dist = lm(min_dist~ tOe + tOa

+ + zonal_dNBR, data = tBF_plot)

> bfmin_dist <- dredge(bmin_dist, beta = "p", extra = list(

+ "R^2", "*" = function(x) {

+ s <- summary(x)

+ c(Rsq = s$r.squared, adjRsq = s$adj.r.squared,

+ F = s$fstatistic[[1]])

+ })

+ )

Fixed term is "(Intercept)"

> subset(bfmin_dist, delta < 2)

Global model call: lm(formula = min_dist ~ tOe + tOa + zonal_dNBR, data = tBF_plot)

---

Model selection table

(Int) tOa tOe znl_dNB R^2 *.Rsq *.adjRsq *.F df logLik AICc delta weight

4 0 6.726 -7.158 0.3936 0.3936 0.3178 5.193 4 -75.028 160.9 0.00 0.261

7 0 -7.040 6.561 0.3860 0.3860 0.3093 5.030 4 -75.146 161.1 0.24 0.232

3 0 -8.076 0.2378 0.2378 0.1930 5.305 3 -77.200 162.0 1.09 0.152

8 0 4.819 -6.744 4.570 0.4613 0.4613 0.3536 4.282 5 -73.903 162.4 1.51 0.123

2 0 7.718 0.2172 0.2172 0.1711 4.717 3 -77.454 162.5 1.60 0.118

5 0 7.686 0.2154 0.2154 0.1692 4.666 3 -77.476 162.6 1.64 0.115

Models ranked by AICc(x)

> par(mar = c(3,5,6,4))

> plot(bfmin_dist, labAsExpr = TRUE)

> summary(model.avg(bfmin_dist, subset = delta < 2,revised.var=TRUE))

Call:

model.avg(object = bfmin_dist, subset = delta < 2, revised.var = TRUE)

Component model call:

lm(formula = min_dist ~ <6 unique rhs>, data = tBF_plot)

Component models:

df logLik AICc delta weight

12 4 -75.03 160.91 0.00 0.26

23 4 -75.15 161.15 0.24 0.23

2 3 -77.20 162.00 1.09 0.15

123 5 -73.90 162.42 1.51 0.12

1 3 -77.45 162.51 1.60 0.12

3 3 -77.48 162.55 1.64 0.12

Term codes:

tOa tOe zonal_dNBR

1 2 3

Model-averaged coefficients:

(full average)

Estimate Std. Error Adjusted SE z value Pr(>|z|)

(Intercept) 0.000 0.000 0.000 NA NA

tOa 3.255 4.098 4.215 0.772 0.44

tOe -5.554 4.264 4.433 1.253 0.21

zonal_dNBR 2.967 3.993 4.106 0.723 0.47

(conditional average)

Estimate Std. Error Adjusted SE z value Pr(>|z|)

(Intercept) 0.000 0.000 0.000 NA NA

tOa 6.492 3.531 3.796 1.710 0.0872 .

tOe -7.238 3.392 3.664 1.975 0.0482 *

zonal_dNBR 6.316 3.575 3.839 1.645 0.0999 .

---

Signif. codes: 0 ‘***’ 0.001 ‘**’ 0.01 ‘*’ 0.05 ‘.’ 0.1 ‘ ’ 1

> confint(model.avg(bfmin_dist, subset = delta < 2,revised.var=TRUE))

2.5 % 97.5 %

(Intercept) 0.0000000 0.00000000

tOa -0.9479297 13.93212244

tOe -14.4184701 -0.05657632

zonal_dNBR -1.2087006 13.84043864

> summary(model.avg(bfmin_dist, subset = cumsum(weight) <= .95))

Call:

model.avg(object = bfmin_dist, subset = cumsum(weight) <= 0.95)

Component model call:

lm(formula = min_dist ~ <6 unique rhs>, data = tBF_plot)

Component models:

df logLik AICc delta weight

12 4 -75.03 160.91 0.00 0.26

23 4 -75.15 161.15 0.24 0.23

2 3 -77.20 162.00 1.09 0.15

123 5 -73.90 162.42 1.51 0.12

1 3 -77.45 162.51 1.60 0.12

3 3 -77.48 162.55 1.64 0.12

Term codes:

tOa tOe zonal_dNBR

1 2 3

Model-averaged coefficients:

(full average)

Estimate Std. Error Adjusted SE z value Pr(>|z|)

(Intercept) 0.000 0.000 0.000 NA NA

tOa 3.255 4.098 4.215 0.772 0.44

tOe -5.554 4.264 4.433 1.253 0.21

zonal_dNBR 2.967 3.993 4.106 0.723 0.47

(conditional average)

Estimate Std. Error Adjusted SE z value Pr(>|z|)

(Intercept) 0.000 0.000 0.000 NA NA

tOa 6.492 3.531 3.796 1.710 0.0872 .

tOe -7.238 3.392 3.664 1.975 0.0482 *

zonal_dNBR 6.316 3.575 3.839 1.645 0.0999 .

---

Signif. codes: 0 ‘***’ 0.001 ‘**’ 0.01 ‘*’ 0.05 ‘.’ 0.1 ‘ ’ 1

> summary(get.models(bfmin_dist, 1)[[1]])

Call:

lm(formula = min_dist ~ tOa + tOe + 1, data = tBF_plot)

Residuals:

Min 1Q Median 3Q Max

-22.845 -6.901 -3.752 7.954 30.255

Coefficients:

Estimate Std. Error t value Pr(>|t|)

(Intercept) 83.54 64.63 1.293 0.2145

tOa 35.87 17.69 2.028 0.0596 .

tOe -105.16 48.74 -2.158 0.0465 *

---

Signif. codes: 0 ‘***’ 0.001 ‘**’ 0.01 ‘*’ 0.05 ‘.’ 0.1 ‘ ’ 1

Residual standard error: 13.68 on 16 degrees of freedom

Multiple R-squared: 0.3936, Adjusted R-squared: 0.3178

F-statistic: 5.193 on 2 and 16 DF, p-value: 0.01828
