# Supplementary material for: Can Siberian alder N-fixation offset N-loss after severe fire? Quantifying post-fire Siberian alder distribution, growth, and N-fixation in boreal Alaska
Source: PLoS One. 2020 Sep 2;15(9):e0238004. doi: 10.1371/journal.pone.0238004 (PMC7467271; doi:10.1371/journal.pone.0238004)
Supplement: S1 File — (ZIP) [file pone.0238004.s005.zip › AIC_BF_lnbio.docx]

> ## lnbio model in boundary fire

> blnbio = lm(lnbio~avg_moisture + avg_O + slope

+ + soil_pH +soilNP

+ +sum_ann_sr + zonal_dNBR, data = tBF_plot)

> bflnbio <- dredge(blnbio, beta = "p", extra = list(

+ "R^2", "*" = function(x) {

+ s <- summary(x)

+ c(Rsq = s$r.squared, adjRsq = s$adj.r.squared,

+ F = s$fstatistic[[1]])

+ })

+ )

Fixed term is "(Intercept)"

> subset(bflnbio, delta < 2)

Global model call: lm(formula = lnbio ~ avg_moisture + avg_O + slope + soil_pH +

soilNP + sum_ann_sr + zonal_dNBR, data = tBF_plot)

---

Model selection table

(Int) avg_mst avg_O slp sol_pH sNP R^2 *.Rsq *.adjRsq *.F df logLik AICc delta weight

19 0 -7.586 -5.776 0.5513 0.5513 0.4952 9.830 4 -70.934 152.7 0.00 0.279

18 0 -7.212 -7.696 0.5296 0.5296 0.4708 9.007 4 -71.383 153.6 0.90 0.178

20 0 -3.893 -4.586 -6.054 0.6072 0.6072 0.5287 7.730 5 -69.670 154.0 1.23 0.151

11 0 -9.899 5.049 0.5204 0.5204 0.4605 8.682 4 -71.567 154.0 1.26 0.148

3 0 -10.060 0.4205 0.4205 0.3864 12.340 3 -73.365 154.3 1.60 0.125

7 0 -9.337 -4.74 0.5086 0.5086 0.4472 8.280 4 -71.798 154.5 1.73 0.118

Models ranked by AICc(x)

> par(mar = c(3,5,6,4))

> plot(bflnbio, labAsExpr = TRUE)

> summary(model.avg(bflnbio, subset = delta < 2))

Call:

model.avg(object = bflnbio, subset = delta < 2)

Component model call:

lm(formula = lnbio ~ <6 unique rhs>, data = tBF_plot)

Component models:

df logLik AICc delta weight

25 4 -70.93 152.73 0.00 0.28

15 4 -71.38 153.62 0.90 0.18

125 5 -69.67 153.96 1.23 0.15

24 4 -71.57 153.99 1.26 0.15

2 3 -73.36 154.33 1.60 0.13

23 4 -71.80 154.45 1.73 0.12

Term codes:

avg_moisture avg_O slope soil_pH soilNP

1 2 3 4 5

Model-averaged coefficients:

(full average)

Estimate Std. Error Adjusted SE z value Pr(>|z|)

(Intercept) 0.0000 0.0000 0.0000 NA NA

avg_O -6.6403 4.3364 4.4556 1.490 0.136

soilNP -3.8997 3.8227 3.9211 0.995 0.320

avg_moisture -1.8738 3.2342 3.2989 0.568 0.570

soil_pH 0.7495 2.0874 2.1331 0.351 0.725

slope -0.5580 1.8044 1.8473 0.302 0.763

(conditional average)

Estimate Std. Error Adjusted SE z value Pr(>|z|)

(Intercept) 0.000 0.000 0.000 NA NA

avg_O -8.081 3.353 3.538 2.284 0.0224 *

soilNP -6.408 2.818 3.032 2.113 0.0346 *

avg_moisture -5.690 3.170 3.367 1.690 0.0910 .

soil_pH 5.049 2.765 2.991 1.688 0.0913 .

slope -4.740 2.799 3.027 1.566 0.1174

---

Signif. codes: 0 ‘***’ 0.001 ‘**’ 0.01 ‘*’ 0.05 ‘.’ 0.1 ‘ ’ 1

> confint(model.avg(bflnbio, subset = delta < 2))

2.5 % 97.5 %

(Intercept) 0.0000000 0.0000000

avg_O -15.0152095 -1.1471008

soilNP -12.3502020 -0.4650645

avg_moisture -12.2880635 0.9086362

soil_pH -0.8123621 10.9106844

slope -10.6738851 1.1931269

> summary(model.avg(bflnbio, subset = cumsum(weight) <= .95))

Call:

model.avg(object = bflnbio, subset = cumsum(weight) <= 0.95)

Component model call:

lm(formula = lnbio ~ <54 unique rhs>, data = tBF_plot)

Component models:

df logLik AICc delta weight

25 4 -70.93 152.73 0.00 0.12

15 4 -71.38 153.62 0.90 0.08

125 5 -69.67 153.96 1.23 0.07

24 4 -71.57 153.99 1.26 0.07

2 3 -73.36 154.33 1.60 0.06

23 4 -71.80 154.45 1.73 0.05

56 4 -71.99 154.84 2.11 0.04

256 5 -70.18 154.98 2.26 0.04

235 5 -70.44 155.50 2.78 0.03

245 5 -70.46 155.53 2.81 0.03

257 5 -70.50 155.62 2.89 0.03

12 4 -72.48 155.82 3.09 0.03

26 4 -72.61 156.08 3.35 0.02

156 5 -70.74 156.10 3.37 0.02

124 5 -70.97 156.55 3.82 0.02

234 5 -70.98 156.59 3.86 0.02

157 5 -71.19 157.00 4.27 0.01

5 3 -74.80 157.21 4.48 0.01

27 4 -73.18 157.21 4.48 0.01

123 5 -71.34 157.30 4.57 0.01

135 5 -71.36 157.34 4.61 0.01

145 5 -71.37 157.35 4.63 0.01

246 5 -71.45 157.51 4.78 0.01

247 5 -71.56 157.74 5.01 0.01

6 3 -75.08 157.75 5.03 0.01

1245 6 -69.45 157.90 5.17 0.01

237 5 -71.67 157.95 5.22 0.01

236 5 -71.68 157.97 5.24 0.01

1256 6 -69.49 157.98 5.25 0.01

1 3 -75.19 157.99 5.26 0.01

1235 6 -69.52 158.03 5.31 0.01

1257 6 -69.53 158.06 5.33 0.01

567 5 -71.88 158.37 5.64 0.01

57 4 -73.81 158.47 5.75 0.01

456 5 -71.94 158.49 5.76 0.01

356 5 -71.98 158.58 5.85 0.01

126 5 -72.22 159.05 6.32 0.01

2567 6 -70.05 159.11 6.38 0.01

2356 6 -70.08 159.16 6.43 0.00

2456 6 -70.08 159.16 6.43 0.00

16 4 -74.15 159.16 6.44 0.00

2357 6 -70.11 159.22 6.49 0.00

14 4 -74.19 159.24 6.52 0.00

2345 6 -70.18 159.36 6.64 0.00

127 5 -72.45 159.51 6.79 0.00

13 4 -74.34 159.53 6.81 0.00

2457 6 -70.27 159.55 6.82 0.00

35 4 -74.46 159.78 7.05 0.00

267 5 -72.60 159.81 7.08 0.00

1234 6 -70.57 160.14 7.42 0.00

45 4 -74.70 160.26 7.53 0.00

36 4 -74.71 160.28 7.56 0.00

46 4 -74.73 160.32 7.60 0.00

1567 6 -70.70 160.40 7.67 0.00

Term codes:

avg_moisture avg_O slope soil_pH soilNP sum_ann_sr zonal_dNBR

1 2 3 4 5 6 7

Model-averaged coefficients:

(full average)

Estimate Std. Error Adjusted SE z value Pr(>|z|)

(Intercept) 0.0000 0.0000 0.0000 NA NA

avg_O -5.4453 4.3970 4.5100 1.207 0.227

soilNP -3.8491 3.8479 3.9580 0.972 0.331

avg_moisture -1.6970 3.0820 3.1599 0.537 0.591

soil_pH 0.7229 2.0666 2.1398 0.338 0.736

slope -0.5819 1.8537 1.9278 0.302 0.763

sum_ann_sr 0.9296 2.4225 2.4918 0.373 0.709

zonal_dNBR -0.2082 1.2055 1.2833 0.162 0.871

(conditional average)

Estimate Std. Error Adjusted SE z value Pr(>|z|)

(Intercept) 0.000 0.000 0.000 NA NA

avg_O -7.493 3.356 3.556 2.107 0.0351 *

soilNP -6.202 3.044 3.263 1.900 0.0574 .

avg_moisture -5.002 3.386 3.591 1.393 0.1637

soil_pH 3.448 3.313 3.528 0.977 0.3284

slope -3.103 3.241 3.463 0.896 0.3703

sum_ann_sr 4.097 3.590 3.793 1.080 0.2801

zonal_dNBR -1.683 3.044 3.291 0.511 0.6092

---

Signif. codes: 0 ‘***’ 0.001 ‘**’ 0.01 ‘*’ 0.05 ‘.’ 0.1 ‘ ’ 1

> summary(get.models(bflnbio, 1)[[1]])

Call:

lm(formula = lnbio ~ avg_O + soilNP + 1, data = tBF_plot)

Residuals:

Min 1Q Median 3Q Max

-15.370 -7.513 -3.369 9.375 16.558

Coefficients:

Estimate Std. Error t value Pr(>|t|)

(Intercept) 50.1805 8.0983 6.196 1.28e-05 ***

avg_O -1.4081 0.4964 -2.836 0.0119 *

soilNP -3.9352 1.8220 -2.160 0.0463 *

---

Signif. codes: 0 ‘***’ 0.001 ‘**’ 0.01 ‘*’ 0.05 ‘.’ 0.1 ‘ ’ 1

Residual standard error: 11.03 on 16 degrees of freedom

Multiple R-squared: 0.5513, Adjusted R-squared: 0.4952

F-statistic: 9.83 on 2 and 16 DF, p-value: 0.001643
