# Supplementary material for: Can Siberian alder N-fixation offset N-loss after severe fire? Quantifying post-fire Siberian alder distribution, growth, and N-fixation in boreal Alaska
Source: PLoS One. 2020 Sep 2;15(9):e0238004. doi: 10.1371/journal.pone.0238004 (PMC7467271; doi:10.1371/journal.pone.0238004)
Supplement: S1 File — (ZIP) [file pone.0238004.s005.zip › AIC_BF_nodule_nfix.docx]

> ## N-fix in BF

> nfix = lm(NFIX~ soilCN + soil_BD + slope + zonal_dNBR , data = tBF_plot)

> bfnfix <- dredge(nfix, beta = "p", extra = list(

+ "R^2", "*" = function(x) {

+ s <- summary(x)

+ c(Rsq = s$r.squared, adjRsq = s$adj.r.squared,

+ F = s$fstatistic[[1]])

+ })

+ )

Fixed term is "(Intercept)"

> subset(bfnfix, delta < 2)

Global model call: lm(formula = NFIX ~ soilCN + soil_BD + slope + zonal_dNBR, data = tBF_plot)

---

Model selection table

(Int) slp sol_BD sCN znl_dNB R^2 *.Rsq *.adjRsq *.F df logLik AICc delta weight

13 0 -2.880 1.858 0.3459 0.3459 0.2641 4.230 4 -53.556 118.0 0.00 0.183

5 0 -2.432 0.2230 0.2230 0.1773 4.879 3 -55.192 118.0 0.01 0.181

2 0 -2.349 0.2080 0.2080 0.1614 4.465 3 -55.374 118.3 0.38 0.151

3 0 2.098 0.1660 0.1660 0.1169 3.383 3 -55.865 119.3 1.36 0.093

4 0 -1.873 1.525 0.2908 0.2908 0.2022 3.281 4 -54.324 119.5 1.54 0.085

10 0 -2.583 1.499 0.2880 0.2880 0.1991 3.237 4 -54.362 119.6 1.61 0.082

6 0 -1.350 -1.498 0.2878 0.2878 0.1988 3.234 4 -54.364 119.6 1.62 0.081

14 0 -1.394 -1.916 1.917 0.4110 0.4110 0.2932 3.489 5 -52.560 119.7 1.77 0.076

1 0 0.0000 0.0000 0.0000 2 -57.589 119.9 1.96 0.069

Models ranked by AICc(x)

> par(mar = c(3,5,6,4))

> plot(bfnfix, labAsExpr = TRUE)

> summary(model.avg(bfnfix, subset = delta < 2))

Call:

model.avg(object = bfnfix, subset = delta < 2)

Component model call:

lm(formula = NFIX ~ <9 unique rhs>, data = tBF_plot)

Component models:

df logLik AICc delta weight

34 4 -53.56 117.97 0.00 0.18

3 3 -55.19 117.98 0.01 0.18

1 3 -55.37 118.35 0.38 0.15

2 3 -55.87 119.33 1.36 0.09

12 4 -54.32 119.51 1.54 0.08

14 4 -54.36 119.58 1.61 0.08

13 4 -54.36 119.59 1.62 0.08

134 5 -52.56 119.74 1.77 0.08

(Null) 2 -57.59 119.93 1.96 0.07

Term codes:

slope soil_BD soilCN zonal_dNBR

1 2 3 4

Model-averaged coefficients:

(full average)

Estimate Std. Error Adjusted SE z value Pr(>|z|)

(Intercept) 0.0000 0.0000 0.0000 NA NA

soilCN -1.2341 1.4648 1.4999 0.823 0.411

zonal_dNBR 0.6068 1.0604 1.0925 0.555 0.579

slope -0.9404 1.2934 1.3310 0.707 0.480

soil_BD 0.3235 0.8521 0.8736 0.370 0.711

(conditional average)

Estimate Std. Error Adjusted SE z value Pr(>|z|)

(Intercept) 0.000 0.000 0.000 NA NA

soilCN -2.368 1.196 1.277 1.854 0.0637 .

zonal_dNBR 1.785 1.097 1.186 1.505 0.1324

slope -1.981 1.210 1.292 1.533 0.1253

soil_BD 1.824 1.165 1.251 1.458 0.1448

---

Signif. codes: 0 ‘***’ 0.001 ‘**’ 0.01 ‘*’ 0.05 ‘.’ 0.1 ‘ ’ 1

> confint(model.avg(bfnfix, subset = delta < 2))

2.5 % 97.5 %

(Intercept) 0.0000000 0.0000000

soilCN -4.8715960 0.1349246

zonal_dNBR -0.5403638 4.1105155

slope -4.5138755 0.5521756

soil_BD -0.6278144 4.2764714

> model.avg(bfnfix, subset = cumsum(weight) <= .95)

Call:

model.avg(object = bfnfix, subset = cumsum(weight) <= 0.95)

Component models:

‘34’ ‘3’ ‘1’ ‘2’ ‘12’ ‘14’ ‘13’ ‘134’ ‘(Null)’ ‘23’ ‘234’ ‘4’

Coefficients:

(Intercept) soilCN zonal_dNBR slope soil_BD

full 0 -1.239876 0.6237194 -0.8532499 0.3224872

subset 0 -2.291888 1.7391815 -1.9808500 1.4076006

> summary(get.models(bfnfix, 1)[[1]])

Call:

lm(formula = NFIX ~ soilCN + zonal_dNBR + 1, data = tBF_plot)

Residuals:

Min 1Q Median 3Q Max

-5.226 -2.683 -1.419 1.919 10.503

Coefficients:

Estimate Std. Error t value Pr(>|t|)

(Intercept) 24.918291 5.907547 4.218 0.000653 ***

soilCN -0.859403 0.319714 -2.688 0.016163 *

zonal_dNBR 0.008584 0.004950 1.734 0.102152

---

Signif. codes: 0 ‘***’ 0.001 ‘**’ 0.01 ‘*’ 0.05 ‘.’ 0.1 ‘ ’ 1

Residual standard error: 4.418 on 16 degrees of freedom

Multiple R-squared: 0.3459, Adjusted R-squared: 0.2641

F-statistic: 4.23 on 2 and 16 DF, p-value: 0.03351
