# Supplementary material for: Can Siberian alder N-fixation offset N-loss after severe fire? Quantifying post-fire Siberian alder distribution, growth, and N-fixation in boreal Alaska
Source: PLoS One. 2020 Sep 2;15(9):e0238004. doi: 10.1371/journal.pone.0238004 (PMC7467271; doi:10.1371/journal.pone.0238004)
Supplement: S1 File — (ZIP) [file pone.0238004.s005.zip › AIC_BF_PCA1.docx]

> ## factor 1 model for growth in BF

> global.model = lm(FAC1_2~ avg_moisture + tsoil_P

+ + soilNP + avg_O + soil_pH + sum_ann_sr

+ + slope + zonal_dNBR, data = tBF_plot)

> dd <- dredge(global.model, beta = "p", extra = list(

+ "R^2", "*" = function(x) {

+ s <- summary(x)

+ c(Rsq = s$r.squared, adjRsq = s$adj.r.squared,

+ F = s$fstatistic[[1]])

+ })

+ )

Fixed term is "(Intercept)"

> subset(dd, delta < 2)

Global model call: lm(formula = FAC1_2 ~ avg_moisture + tsoil_P + soilNP + avg_O +

soil_pH + sum_ann_sr + slope + zonal_dNBR, data = tBF_plot)

---

Model selection table

(Int) avg_mst avg_O sol_pH R^2 *.Rsq *.adjRsq *.F df logLik AICc delta weight

12 0 -0.3051 -0.4441 0.3823 0.8028 0.8028 0.7633 20.35 5 -10.797 36.2 0 1

Models ranked by AICc(x)

> par(mar = c(3,5,6,4))

> plot(dd, labAsExpr = TRUE)

> summary(model.avg(dd, subset = delta < 2))

Error in model.avg.model.selection(dd, subset = delta < 2) :

'object' consists of only one model

> confint(model.avg(dd, subset = delta < 2))

Error in model.avg.model.selection(dd, subset = delta < 2) :

'object' consists of only one model

> summary(model.avg(dd, subset = cumsum(weight) <= .95))

Call:

model.avg(object = dd, subset = cumsum(weight) <= 0.95)

Component model call:

lm(formula = FAC1_2 ~ <76 unique rhs>, data = tBF_plot)

Component models:

df logLik AICc delta weight

124 5 -10.80 36.21 0.00 0.26

1245 6 -9.73 38.47 2.26 0.08

125 5 -12.21 39.03 2.82 0.06

24 4 -14.19 39.24 3.03 0.06

1247 6 -10.47 39.95 3.74 0.04

1246 6 -10.70 40.40 4.19 0.03

1248 6 -10.77 40.53 4.33 0.03

1234 6 -10.79 40.57 4.36 0.03

156 5 -13.03 40.67 4.46 0.03

1256 6 -11.04 41.08 4.87 0.02

246 5 -13.24 41.10 4.89 0.02

15 4 -15.16 41.18 4.97 0.02

256 5 -13.74 42.10 5.89 0.01

17 4 -15.66 42.17 5.96 0.01

12 4 -15.70 42.25 6.04 0.01

157 5 -13.85 42.31 6.10 0.01

245 5 -13.85 42.32 6.11 0.01

234 5 -13.99 42.60 6.39 0.01

1258 6 -11.83 42.66 6.45 0.01

147 5 -14.05 42.71 6.50 0.01

247 5 -14.07 42.76 6.55 0.01

127 5 -14.10 42.81 6.60 0.01

248 5 -14.11 42.83 6.62 0.01

1257 6 -11.97 42.93 6.72 0.01

12456 7 -9.46 43.11 6.90 0.01

1235 6 -12.18 43.35 7.14 0.01

26 4 -16.25 43.36 7.15 0.01

145 5 -14.40 43.42 7.21 0.01

12345 7 -9.68 43.54 7.33 0.01

12457 7 -9.68 43.55 7.34 0.01

12458 7 -9.72 43.63 7.42 0.01

126 5 -14.57 43.76 7.55 0.01

56 4 -16.55 43.95 7.74 0.01

2456 6 -12.51 44.02 7.81 0.01

1356 6 -12.54 44.09 7.88 0.01

158 5 -14.74 44.10 7.90 0.01

123 5 -14.78 44.17 7.96 0.00

25 4 -16.73 44.31 8.10 0.00

1456 6 -12.78 44.56 8.35 0.00

1567 6 -12.84 44.67 8.46 0.00

178 5 -15.06 44.74 8.53 0.00

14 4 -16.95 44.75 8.54 0.00

135 5 -15.15 44.91 8.70 0.00

1568 6 -12.97 44.93 8.72 0.00

12467 7 -10.46 45.10 8.89 0.00

12347 7 -10.46 45.11 8.90 0.00

167 5 -15.25 45.12 8.91 0.00

12478 7 -10.47 45.13 8.92 0.00

1457 6 -13.11 45.22 9.01 0.00

137 5 -15.32 45.25 9.04 0.00

1578 6 -13.14 45.28 9.07 0.00

16 4 -17.24 45.34 9.13 0.00

2 3 -18.92 45.43 9.22 0.00

2346 6 -13.22 45.45 9.24 0.00

12468 7 -10.64 45.45 9.24 0.00

2467 6 -13.24 45.48 9.27 0.00

2468 6 -13.24 45.49 9.28 0.00

23 4 -17.33 45.52 9.31 0.00

12346 7 -10.70 45.58 9.37 0.00

12348 7 -10.76 45.70 9.49 0.00

128 5 -15.57 45.76 9.55 0.00

146 5 -15.65 45.91 9.70 0.00

258 5 -15.65 45.91 9.71 0.00

1237 6 -13.48 45.97 9.76 0.00

2568 6 -13.50 46.01 9.80 0.00

12568 7 -10.94 46.05 9.84 0.00

12356 7 -10.94 46.06 9.86 0.00

2567 6 -13.59 46.18 9.97 0.00

2458 6 -13.61 46.21 10.00 0.00

12567 7 -11.04 46.26 10.05 0.00

236 5 -15.84 46.30 10.09 0.00

1278 6 -13.68 46.35 10.15 0.00

27 4 -17.76 46.38 10.17 0.00

2356 6 -13.73 46.46 10.26 0.00

2345 6 -13.76 46.51 10.31 0.00

1267 6 -13.78 46.56 10.35 0.00

Term codes:

avg_moisture avg_O slope soil_pH soilNP sum_ann_sr tsoil_P zonal_dNBR

1 2 3 4 5 6 7 8

Model-averaged coefficients:

(full average)

Estimate Std. Error Adjusted SE z value Pr(>|z|)

(Intercept) 0.000000 0.000000 0.000000 NA NA

avg_moisture -0.285511 0.194558 0.201084 1.420 0.156

avg_O -0.363030 0.220941 0.226967 1.599 0.110

soil_pH 0.234798 0.198814 0.204040 1.151 0.250

soilNP -0.104560 0.169112 0.172901 0.605 0.545

tsoil_P 0.026108 0.096418 0.099250 0.263 0.793

sum_ann_sr 0.040137 0.113056 0.116288 0.345 0.730

zonal_dNBR -0.003597 0.047756 0.051234 0.070 0.944

slope -0.003116 0.048587 0.051974 0.060 0.952

(conditional average)

Estimate Std. Error Adjusted SE z value Pr(>|z|)

(Intercept) 0.00000 0.00000 0.00000 NA NA

avg_moisture -0.34980 0.15456 0.16447 2.127 0.0334 *

avg_O -0.42476 0.17577 0.18453 2.302 0.0213 *

soil_pH 0.34281 0.14382 0.15413 2.224 0.0261 *

soilNP -0.27515 0.16829 0.17813 1.545 0.1224

tsoil_P 0.17285 0.19022 0.19963 0.866 0.3866

sum_ann_sr 0.19679 0.17844 0.18835 1.045 0.2961

zonal_dNBR -0.03896 0.15273 0.16448 0.237 0.8127

slope -0.03422 0.15766 0.16911 0.202 0.8396

---

Signif. codes: 0 ‘***’ 0.001 ‘**’ 0.01 ‘*’ 0.05 ‘.’ 0.1 ‘ ’ 1

> summary(get.models(dd, 1)[[1]])

Call:

lm(formula = FAC1_2 ~ avg_moisture + avg_O + soil_pH + 1, data = tBF_plot)

Residuals:

Min 1Q Median 3Q Max

-0.77808 -0.26166 -0.01907 0.43371 0.62620

Coefficients:

Estimate Std. Error t value Pr(>|t|)

(Intercept) -2.49448 1.45235 -1.718 0.10645

avg_moisture -0.09745 0.03838 -2.539 0.02269 *

avg_O -0.08928 0.02416 -3.696 0.00216 **

soil_pH 1.01432 0.31885 3.181 0.00620 **

---

Signif. codes: 0 ‘***’ 0.001 ‘**’ 0.01 ‘*’ 0.05 ‘.’ 0.1 ‘ ’ 1

Residual standard error: 0.4807 on 15 degrees of freedom

Multiple R-squared: 0.8028, Adjusted R-squared: 0.7633

F-statistic: 20.35 on 3 and 15 DF, p-value: 1.518e-05
