# Supplementary material for: Can Siberian alder N-fixation offset N-loss after severe fire? Quantifying post-fire Siberian alder distribution, growth, and N-fixation in boreal Alaska
Source: PLoS One. 2020 Sep 2;15(9):e0238004. doi: 10.1371/journal.pone.0238004 (PMC7467271; doi:10.1371/journal.pone.0238004)
Supplement: S1 File — (ZIP) [file pone.0238004.s005.zip › AIC_BF_PCA2.docx]

> ## factor 2 model for growth in BF

> factor2.BF = lm(FAC2_2~ elev + soil_pH + zonal_dNBR , data = tBF_plot)

> BFFAC2 <- dredge(factor2.BF, beta = "p", extra = list(

+ "R^2", "*" = function(x) {

+ s <- summary(x)

+ c(Rsq = s$r.squared, adjRsq = s$adj.r.squared,

+ F = s$fstatistic[[1]])

+ })

+ )

Fixed term is "(Intercept)"

> subset(BFFAC2, delta < 2)

Global model call: lm(formula = FAC2_2 ~ elev + soil_pH + zonal_dNBR, data = tBF_plot)

---

Model selection table

(Int) elv sol_pH R^2 *.Rsq *.adjRsq *.F df logLik AICc delta weight

4 0 0.3417 0.2867 0.5922 0.5922 0.5412 11.62 4 -5.94 22.7 0 1

Models ranked by AICc(x)

> par(mar = c(3,5,6,4))

> plot(BFFAC2, labAsExpr = TRUE)

> summary(model.avg(BFFAC2, subset = delta < 2))

Error in model.avg.model.selection(BFFAC2, subset = delta < 2) :

'object' consists of only one model

> confint(model.avg(BFFAC2, subset = delta < 2))

Error in model.avg.model.selection(BFFAC2, subset = delta < 2) :

'object' consists of only one model

> model.avg(BFFAC2, subset = cumsum(weight) <= .95)

Call:

model.avg(object = BFFAC2, subset = cumsum(weight) <= 0.95)

Component models:

‘12’ ‘123’

Coefficients:

(Intercept) elev soil_pH zonal_dNBR

full 0 0.3429229 0.2858892 -0.0006123515

subset 0 0.3429229 0.2858892 -0.0046156326

> summary(get.models(BFFAC2, 1)[[1]])

Call:

lm(formula = FAC2_2 ~ elev + soil_pH + 1, data = tBF_plot)

Residuals:

Min 1Q Median 3Q Max

-0.54838 -0.21112 -0.02089 0.16533 0.61200

Coefficients:

Estimate Std. Error t value Pr(>|t|)

(Intercept) -5.758083 1.184037 -4.863 0.000173 ***

elev 0.004441 0.001136 3.908 0.001252 **

soil_pH 0.773583 0.235896 3.279 0.004720 **

---

Signif. codes: 0 ‘***’ 0.001 ‘**’ 0.01 ‘*’ 0.05 ‘.’ 0.1 ‘ ’ 1

Residual standard error: 0.3605 on 16 degrees of freedom

Multiple R-squared: 0.5922, Adjusted R-squared: 0.5412

F-statistic: 11.62 on 2 and 16 DF, p-value: 0.0007654
