# Supplementary material for: Can Siberian alder N-fixation offset N-loss after severe fire? Quantifying post-fire Siberian alder distribution, growth, and N-fixation in boreal Alaska
Source: PLoS One. 2020 Sep 2;15(9):e0238004. doi: 10.1371/journal.pone.0238004 (PMC7467271; doi:10.1371/journal.pone.0238004)
Supplement: S1 File — (ZIP) [file pone.0238004.s005.zip › AIC_BF_plant_nfix.docx]

> ## pynifx in BF

> pynfix = lm(PYNFIX ~ avg_O + soilNP

+ + slope + sum_ann_sr + zonal_dNBR

+ , data = tBF_plot)

> bfpynfix <- dredge(pynfix, beta = "p", extra = list(

+ "R^2", "*" = function(x) {

+ s <- summary(x)

+ c(Rsq = s$r.squared, adjRsq = s$adj.r.squared,

+ F = s$fstatistic[[1]])

+ })

+ )

Fixed term is "(Intercept)"

> subset(bfpynfix, delta < 2)

Global model call: lm(formula = PYNFIX ~ avg_O + soilNP + slope + sum_ann_sr + zonal_dNBR,

data = tBF_plot)

---

Model selection table

(Int) avg_O slp sNP R^2 *.Rsq *.adjRsq *.F df logLik AICc delta weight

6 0 -2.832 -3.204 0.5222 0.5222 0.4625 8.743 4 -56.884 124.6 0.00 0.555

4 0 -3.704 -2.767 0.4744 0.4744 0.4087 7.219 4 -57.790 126.4 1.81 0.224

5 0 -4.398 0.3752 0.3752 0.3385 10.210 3 -59.431 126.5 1.84 0.221

Models ranked by AICc(x)

> par(mar = c(3,5,6,4))

> plot(bfpynfix, labAsExpr = TRUE)

> summary(model.avg(bfpynfix, subset = delta < 2))

Call:

model.avg(object = bfpynfix, subset = delta < 2)

Component model call:

lm(formula = PYNFIX ~ <3 unique rhs>, data = tBF_plot)

Component models:

df logLik AICc delta weight

13 4 -56.88 124.62 0.00 0.55

12 4 -57.79 126.44 1.81 0.22

3 3 -59.43 126.46 1.84 0.22

Term codes:

avg_O slope soilNP

1 2 3

Model-averaged coefficients:

(full average)

Estimate Std. Error Adjusted SE z value Pr(>|z|)

(Intercept) 0.000 0.000 0.000 NA NA

avg_O -2.401 1.751 1.813 1.324 0.185

soilNP -2.750 1.932 1.988 1.383 0.167

slope -0.620 1.316 1.342 0.462 0.644

(conditional average)

Estimate Std. Error Adjusted SE z value Pr(>|z|)

(Intercept) 0.000 0.000 0.000 NA NA

avg_O -3.083 1.354 1.455 2.119 0.0341 *

soilNP -3.545 1.413 1.510 2.348 0.0189 *

slope -2.767 1.339 1.448 1.910 0.0561 .

---

Signif. codes: 0 ‘***’ 0.001 ‘**’ 0.01 ‘*’ 0.05 ‘.’ 0.1 ‘ ’ 1

> confint(model.avg(bfpynfix, subset = delta < 2))

2.5 % 97.5 %

(Intercept) 0.000000 0.0000000

avg_O -5.934956 -0.2308260

soilNP -6.503748 -0.5852914

slope -5.605476 0.0718415

> model.avg(bfpynfix, subset = cumsum(weight) <= .95)

Call:

model.avg(object = bfpynfix, subset = cumsum(weight) <= 0.95)

Component models:

‘13’ ‘12’ ‘3’ ‘123’ ‘34’ ‘1’ ‘134’ ‘135’ ‘23’ ‘35’ ‘124’ ‘125’ ‘14’ ‘2’ ‘234’ ‘15’ ‘345’ ‘1235’

Coefficients:

(Intercept) avg_O soilNP slope sum_ann_sr zonal_dNBR

full 0 -2.201618 -2.532909 -0.7003994 0.2345651 0.002297737

subset 0 -3.110083 -3.369947 -2.1470888 1.2832402 0.019044536

> summary(get.models(bfpynfix, 1)[[1]])

Call:

lm(formula = PYNFIX ~ avg_O + soilNP + 1, data = tBF_plot)

Residuals:

Min 1Q Median 3Q Max

-6.899 -3.861 -0.519 4.429 10.730

Coefficients:

Estimate Std. Error t value Pr(>|t|)

(Intercept) 22.2143 3.8657 5.746 3e-05 ***

avg_O -0.5257 0.2370 -2.218 0.0414 *

soilNP -2.1827 0.8697 -2.510 0.0232 *

---

Signif. codes: 0 ‘***’ 0.001 ‘**’ 0.01 ‘*’ 0.05 ‘.’ 0.1 ‘ ’ 1

Residual standard error: 5.264 on 16 degrees of freedom

Multiple R-squared: 0.5222, Adjusted R-squared: 0.4625

F-statistic: 8.743 on 2 and 16 DF, p-value: 0.002717
