# Supplementary material for: Can Siberian alder N-fixation offset N-loss after severe fire? Quantifying post-fire Siberian alder distribution, growth, and N-fixation in boreal Alaska
Source: PLoS One. 2020 Sep 2;15(9):e0238004. doi: 10.1371/journal.pone.0238004 (PMC7467271; doi:10.1371/journal.pone.0238004)
Supplement: S1 File — (ZIP) [file pone.0238004.s005.zip › AIC_regional_density.docx]

> ## min_dist model in study area

> smin_dist = lm(min_dist~ soilCN + tavg_moisture

+ + fire_id, data = tBothFires_plot)

> samin_dist <- dredge(smin_dist, beta = "p", extra = list(

+ "R^2", "*" = function(x) {

+ s <- summary(x)

+ c(Rsq = s$r.squared, adjRsq = s$adj.r.squared,

+ F = s$fstatistic[[1]])

+ })

+ )

Fixed term is "(Intercept)"

> subset(samin_dist, delta < 2)

Global model call: lm(formula = min_dist ~ soilCN + tavg_moisture + fire_id, data = tBothFires_plot)

---

Model selection table

(Int) sCN tvg_mst R^2 *.Rsq *.adjRsq *.F df logLik AICc delta weight

5 0 -7.046 0.1683 0.1683 0.1464 7.690 3 -166.302 339.3 0.00 0.619

7 0 3.05 -6.089 0.1990 0.1990 0.1557 4.597 4 -165.550 340.2 0.97 0.381

Models ranked by AICc(x)

> par(mar = c(3,5,6,4))

> plot(samin_dist, labAsExpr = TRUE)

> summary(model.avg(samin_dist, subset = delta < 2))

Call:

model.avg(object = samin_dist, subset = delta < 2)

Component model call:

lm(formula = min_dist ~ <2 unique rhs>, data = tBothFires_plot)

Component models:

df logLik AICc delta weight

2 3 -166.30 339.27 0.00 0.62

12 4 -165.55 340.24 0.97 0.38

Term codes:

soilCN tavg_moisture

1 2

Model-averaged coefficients:

(full average)

Estimate Std. Error Adjusted SE z value Pr(>|z|)

(Intercept) 0.000 0.000 0.000 NA NA

tavg_moisture -6.682 2.590 2.673 2.499 0.0124 *

soilCN 1.162 2.166 2.205 0.527 0.5982

(conditional average)

Estimate Std. Error Adjusted SE z value Pr(>|z|)

(Intercept) 0.000 0.000 0.000 NA NA

tavg_moisture -6.682 2.590 2.673 2.499 0.0124 *

soilCN 3.050 2.560 2.646 1.152 0.2492

---

Signif. codes: 0 ‘***’ 0.001 ‘**’ 0.01 ‘*’ 0.05 ‘.’ 0.1 ‘ ’ 1

> confint(model.avg(samin_dist, subset = delta < 2))

2.5 % 97.5 %

(Intercept) 0.00000 0.000000

tavg_moisture -11.92141 -1.441655

soilCN -2.13733 8.236614

> summary(model.avg(samin_dist, subset = cumsum(weight) <= .95))

Call:

model.avg(object = samin_dist, subset = cumsum(weight) <= 0.95)

Component model call:

lm(formula = min_dist ~ <5 unique rhs>, data = tBothFires_plot)

Component models:

df logLik AICc delta weight

3 3 -166.30 339.27 0.00 0.44

23 4 -165.55 340.24 0.97 0.27

13 4 -166.14 341.43 2.16 0.15

123 5 -165.51 342.79 3.51 0.08

2 3 -168.40 343.46 4.19 0.05

Term codes:

fire_id soilCN tavg_moisture

1 2 3

Model-averaged coefficients:

(full average)

Estimate Std. Error Adjusted SE z value Pr(>|z|)

(Intercept) 0.0000 0.0000 0.0000 NA NA

tavg_moisture -6.3142 2.9505 3.0212 2.090 0.0366 *

soilCN 1.3105 2.3209 2.3608 0.555 0.5788

fire_idWDF -0.2672 1.3480 1.3873 0.193 0.8473

(conditional average)

Estimate Std. Error Adjusted SE z value Pr(>|z|)

(Intercept) 0.000 0.000 0.000 NA NA

tavg_moisture -6.680 2.601 2.686 2.487 0.0129 *

soilCN 3.238 2.659 2.744 1.180 0.2381

fire_idWDF -1.174 2.630 2.718 0.432 0.6659

---

Signif. codes: 0 ‘***’ 0.001 ‘**’ 0.01 ‘*’ 0.05 ‘.’ 0.1 ‘ ’ 1

> summary(get.models(samin_dist, 1)[[1]])

Call:

lm(formula = min_dist ~ tavg_moisture + 1, data = tBothFires_plot)

Residuals:

Min 1Q Median 3Q Max

-22.468 -11.905 -4.437 11.840 36.570

Coefficients:

Estimate Std. Error t value Pr(>|t|)

(Intercept) 56.47 14.87 3.798 0.000512 ***

tavg_moisture -72.23 26.05 -2.773 0.008557 **

---

Signif. codes: 0 ‘***’ 0.001 ‘**’ 0.01 ‘*’ 0.05 ‘.’ 0.1 ‘ ’ 1

Residual standard error: 15.87 on 38 degrees of freedom

Multiple R-squared: 0.1683, Adjusted R-squared: 0.1464

F-statistic: 7.69 on 1 and 38 DF, p-value: 0.008557
