# Supplementary material for: Can Siberian alder N-fixation offset N-loss after severe fire? Quantifying post-fire Siberian alder distribution, growth, and N-fixation in boreal Alaska
Source: PLoS One. 2020 Sep 2;15(9):e0238004. doi: 10.1371/journal.pone.0238004 (PMC7467271; doi:10.1371/journal.pone.0238004)
Supplement: S1 File — (ZIP) [file pone.0238004.s005.zip › AIC_regional_lnbio.docx]

> ## lnbio model in study area

> slnbio = lm(lnbio~soilNP + tavg_O + fire_id , data = tBothFires_plot)

> salnbio <- dredge(slnbio, beta = "p", extra = list(

+ "R^2", "*" = function(x) {

+ s <- summary(x)

+ c(Rsq = s$r.squared, adjRsq = s$adj.r.squared,

+ F = s$fstatistic[[1]])

+ })

+ )

Fixed term is "(Intercept)"

> subset(salnbio, delta < 2)

Global model call: lm(formula = lnbio ~ soilNP + tavg_O + fire_id, data = tBothFires_plot)

---

Model selection table

(Int) fir_id tvg_O R^2 *.Rsq *.adjRsq *.F df logLik AICc delta weight

5 0 7.262 0.3517 0.3517 0.3347 20.62 3 -147.79 302.2 0.00 0.544

6 0 + 6.854 0.3852 0.3852 0.3520 11.59 4 -146.73 302.6 0.36 0.456

Models ranked by AICc(x)

> par(mar = c(3,5,6,4))

> plot(salnbio, labAsExpr = TRUE)

> summary(model.avg(salnbio, subset = delta < 2))

Call:

model.avg(object = salnbio, subset = delta < 2)

Component model call:

lm(formula = lnbio ~ <2 unique rhs>, data = tBothFires_plot)

Component models:

df logLik AICc delta weight

2 3 -147.79 302.25 0.00 0.54

12 4 -146.73 302.60 0.36 0.46

Term codes:

fire_id tavg_O

1 2

Model-averaged coefficients:

(full average)

Estimate Std. Error Adjusted SE z value Pr(>|z|)

(Intercept) 0.000 0.000 0.000 NA NA

tavg_O 7.077 1.612 1.665 4.250 2.14e-05 ***

fire_idWDF -1.034 1.563 1.589 0.651 0.515

(conditional average)

Estimate Std. Error Adjusted SE z value Pr(>|z|)

(Intercept) 0.000 0.000 0.000 NA NA

tavg_O 7.077 1.612 1.665 4.250 2.14e-05 ***

fire_idWDF -2.270 1.599 1.653 1.373 0.17

---

Signif. codes: 0 ‘***’ 0.001 ‘**’ 0.01 ‘*’ 0.05 ‘.’ 0.1 ‘ ’ 1

> confint(model.avg(salnbio, subset = delta < 2))

2.5 % 97.5 %

(Intercept) 0.000000 0.0000000

tavg_O 3.813102 10.3399031

fire_idWDF -5.510161 0.9703103

> summary(model.avg(salnbio, subset = cumsum(weight) <= .95))

Call:

model.avg(object = salnbio, subset = cumsum(weight) <= 0.95)

Component model call:

lm(formula = lnbio ~ <3 unique rhs>, data = tBothFires_plot)

Component models:

df logLik AICc delta weight

3 3 -147.79 302.25 0.00 0.47

13 4 -146.73 302.60 0.36 0.39

23 4 -147.77 304.67 2.43 0.14

Term codes:

fire_id soilNP tavg_O

1 2 3

Model-averaged coefficients:

(full average)

Estimate Std. Error Adjusted SE z value Pr(>|z|)

(Intercept) 0.00000 0.00000 0.00000 NA NA

tavg_O 6.96267 1.64086 1.69329 4.112 3.92e-05 ***

fire_idWDF -0.89044 1.49384 1.51674 0.587 0.557

soilNP -0.04849 0.62404 0.64435 0.075 0.940

(conditional average)

Estimate Std. Error Adjusted SE z value Pr(>|z|)

(Intercept) 0.0000 0.0000 0.0000 NA NA

tavg_O 6.9627 1.6409 1.6933 4.112 3.92e-05 ***

fire_idWDF -2.2699 1.5992 1.6532 1.373 0.170

soilNP -0.3484 1.6411 1.6966 0.205 0.837

---

Signif. codes: 0 ‘***’ 0.001 ‘**’ 0.01 ‘*’ 0.05 ‘.’ 0.1 ‘ ’ 1

> summary(get.models(salnbio, 1)[[1]])

Call:

lm(formula = lnbio ~ tavg_O + 1, data = tBothFires_plot)

Residuals:

Min 1Q Median 3Q Max

-17.799 -6.094 -2.148 4.702 26.596

Coefficients:

Estimate Std. Error t value Pr(>|t|)

(Intercept) -117.66 28.76 -4.092 0.000215 ***

tavg_O 188.18 41.44 4.541 5.51e-05 ***

---

Signif. codes: 0 ‘***’ 0.001 ‘**’ 0.01 ‘*’ 0.05 ‘.’ 0.1 ‘ ’ 1

Residual standard error: 9.989 on 38 degrees of freedom

Multiple R-squared: 0.3517, Adjusted R-squared: 0.3347

F-statistic: 20.62 on 1 and 38 DF, p-value: 5.514e-05
