# Supplementary material for: Can Siberian alder N-fixation offset N-loss after severe fire? Quantifying post-fire Siberian alder distribution, growth, and N-fixation in boreal Alaska
Source: PLoS One. 2020 Sep 2;15(9):e0238004. doi: 10.1371/journal.pone.0238004 (PMC7467271; doi:10.1371/journal.pone.0238004)
Supplement: S1 File — (ZIP) [file pone.0238004.s005.zip › AIC_regional_nodule_nfix.docx]

> ## N-fix in study area

> snfix = lm(NFIX~ fire_id, data = tBothFires_plot)

> sanfix <- dredge(snfix, beta = "p", extra = list(

+ "R^2", "*" = function(x) {

+ s <- summary(x)

+ c(Rsq = s$r.squared, adjRsq = s$adj.r.squared,

+ F = s$fstatistic[[1]])

+ })

+ )

Fixed term is "(Intercept)"

> subset(sanfix, delta < 2)

Global model call: lm(formula = NFIX ~ fire_id, data = tBothFires_plot)

---

Model selection table

(Int) fir_id R^2 *.Rsq *.adjRsq *.F df logLik AICc delta weight

2 0 + 0.3098 0.3098 0.2916 17.05 3 -111.686 230 0 1

Models ranked by AICc(x)

> par(mar = c(3,5,6,4))

> plot(sanfix, labAsExpr = TRUE)

> summary(model.avg(sanfix))

Call:

model.avg(object = sanfix)

Component model call:

lm(formula = NFIX ~ <2 unique rhs>, data = tBothFires_plot)

Component models:

df logLik AICc delta weight

1 3 -111.69 230.04 0.00 1

(Null) 2 -119.10 242.52 12.49 0

Term codes:

fire_id

1

Model-averaged coefficients:

(full average)

Estimate Std. Error Adjusted SE z value Pr(>|z|)

(Intercept) 0.0000 0.0000 0.0000 NA NA

fire_idWDF -2.6733 0.6586 0.6796 3.934 8.36e-05 ***

(conditional average)

Estimate Std. Error Adjusted SE z value Pr(>|z|)

(Intercept) 0.0000 0.0000 0.0000 NA NA

fire_idWDF -2.6785 0.6486 0.6699 3.998 6.38e-05 ***

---

Signif. codes: 0 ‘***’ 0.001 ‘**’ 0.01 ‘*’ 0.05 ‘.’ 0.1 ‘ ’ 1

> confint(model.avg(sanfix))

2.5 % 97.5 %

(Intercept) 0.000000 0.000000

fire_idWDF -3.991531 -1.365461

> model.avg(sanfix, subset = cumsum(weight) <= .95)

Error in model.avg.model.selection(sanfix, subset = cumsum(weight) <= :

'object' consists of only one model

In addition: Warning message:

In min(x) : no non-missing arguments to min; returning Inf

> summary(get.models(sanfix, 1)[[1]])

Call:

lm(formula = NFIX ~ fire_id + 1, data = tBothFires_plot)

Residuals:

Min 1Q Median 3Q Max

-7.2910 -1.8379 -0.4079 1.5444 13.5415

Coefficients:

Estimate Std. Error t value Pr(>|t|)

(Intercept) 12.8768 0.9293 13.86 < 2e-16 ***

fire_idWDF -5.2962 1.2825 -4.13 0.000192 ***

---

Signif. codes: 0 ‘***’ 0.001 ‘**’ 0.01 ‘*’ 0.05 ‘.’ 0.1 ‘ ’ 1

Residual standard error: 4.051 on 38 degrees of freedom

Multiple R-squared: 0.3098, Adjusted R-squared: 0.2916

F-statistic: 17.05 on 1 and 38 DF, p-value: 0.0001919
