# Supplementary material for: Can Siberian alder N-fixation offset N-loss after severe fire? Quantifying post-fire Siberian alder distribution, growth, and N-fixation in boreal Alaska
Source: PLoS One. 2020 Sep 2;15(9):e0238004. doi: 10.1371/journal.pone.0238004 (PMC7467271; doi:10.1371/journal.pone.0238004)
Supplement: S1 File — (ZIP) [file pone.0238004.s005.zip › AIC_regional_PCA1.docx]

> ## factor 1 model for growth in SA

> factor1.SA = lm(FAC1_2~ soilNP + tsoil_pH + tavg_moisture

+ + tavg_O + tsum_ann_sr + tOe + fire_id,

+ data = tBothFires_plot)

> SAFAC1 <- dredge(factor1.SA, beta = "p", extra = list(

+ "R^2", "*" = function(x) {

+ s <- summary(x)

+ c(Rsq = s$r.squared, adjRsq = s$adj.r.squared,

+ F = s$fstatistic[[1]])

+ })

+ )

Fixed term is "(Intercept)"

> subset(SAFAC1, delta < 2)

Global model call: lm(formula = FAC1_2 ~ soilNP + tsoil_pH + tavg_moisture + tavg_O +

tsum_ann_sr + tOe + fire_id, data = tBothFires_plot)

---

Model selection table

(Int) fir_id sNP tvg_O tOe tsl_pH R^2 *.Rsq *.adjRsq *.F df logLik AICc delta weight

27 0 -0.2302 0.6075 0.3090 0.5545 0.5545 0.5174 14.94 5 -40.080 91.9 0.00 0.301

26 0 + 0.6960 0.2747 0.5476 0.5476 0.5099 14.52 5 -40.389 92.5 0.62 0.221

28 0 + -0.1802 0.6358 0.3174 0.5775 0.5775 0.5293 11.96 6 -39.018 92.6 0.66 0.217

25 0 0.6577 0.2488 0.5042 0.5042 0.4774 18.82 4 -42.218 93.6 1.65 0.132

58 0 + 0.6748 0.2667 -0.1436 0.5666 0.5666 0.5171 11.44 6 -39.530 93.6 1.68 0.130

Models ranked by AICc(x)

> par(mar = c(3,5,6,4))

> plot(SAFAC1, labAsExpr = TRUE)

> summary(model.avg(SAFAC1, subset = delta < 2, revised.var=TRUE))

Call:

model.avg(object = SAFAC1, subset = delta < 2, revised.var = TRUE)

Component model call:

lm(formula = FAC1_2 ~ <5 unique rhs>, data = tBothFires_plot)

Component models:

df logLik AICc delta weight

234 5 -40.08 91.92 0.00 0.30

134 5 -40.39 92.54 0.62 0.22

1234 6 -39.02 92.58 0.66 0.22

34 4 -42.22 93.58 1.65 0.13

1345 6 -39.53 93.60 1.68 0.13

Term codes:

fire_id soilNP tavg_O tOe tsoil_pH

1 2 3 4 5

Model-averaged coefficients:

(full average)

Estimate Std. Error Adjusted SE z value Pr(>|z|)

(Intercept) 0.00000 0.00000 0.00000 NA NA

soilNP -0.10830 0.13419 0.13598 0.796 0.4258

tavg_O 0.64852 0.11990 0.12377 5.240 2e-07 ***

tOe 0.28983 0.11770 0.12164 2.383 0.0172 *

fire_idWDF 0.10740 0.12905 0.13113 0.819 0.4128

tsoil_pH -0.01865 0.06381 0.06480 0.288 0.7735

(conditional average)

Estimate Std. Error Adjusted SE z value Pr(>|z|)

(Intercept) 0.0000 0.0000 0.0000 NA NA

soilNP -0.2093 0.1169 0.1208 1.732 0.0833 .

tavg_O 0.6485 0.1199 0.1238 5.240 1.6e-07 ***

tOe 0.2898 0.1177 0.1216 2.383 0.0172 *

fire_idWDF 0.1893 0.1177 0.1217 1.556 0.1198

tsoil_pH -0.1436 0.1158 0.1200 1.197 0.2314

---

Signif. codes: 0 ‘***’ 0.001 ‘**’ 0.01 ‘*’ 0.05 ‘.’ 0.1 ‘ ’ 1

> confint(model.avg(SAFAC1, subset = delta < 2))

2.5 % 97.5 %

(Intercept) 0.00000000 0.00000000

soilNP -0.44608264 0.02758002

tavg_O 0.40593610 0.89111350

tOe 0.05142923 0.52823984

fire_idWDF -0.04921183 0.42776224

tsoil_pH -0.37869795 0.09156841

> model.avg(SAFAC1, subset = cumsum(weight) <= .95)

Call:

model.avg(object = SAFAC1, subset = cumsum(weight) <= 0.95)

Component models:

‘245’ ‘145’ ‘1245’ ‘45’ ‘1456’ ‘456’ ‘2456’ ‘2345’ ‘2457’ ‘1457’ ‘12456’ ‘1345’ ‘12457’ ‘12345’ ‘4’ ‘345’

‘14’ ‘46’ ‘3456’ ‘457’ ‘13456’ ‘14567’ ‘146’ ‘23456’ ‘4567’ ‘24’ ‘346’ ‘24567’ ‘34’ ‘23457’ ‘47’ ‘467’

‘124’ ‘134’ ‘13457’ ‘123456’ ‘124567’ ‘246’ ‘1346’ ‘234’ ‘123457’ ‘3457’

Coefficients:

(Intercept) soilNP tavg_O tOe fire_idWDF tsoil_pH tavg_moisture tsum_ann_sr

full 0 -0.09390231 0.6067255 0.2418043 0.08595808 -0.0427684 0.01676931 -0.0005915361

subset 0 -0.19444757 0.6067255 0.2827756 0.18390557 -0.1348671 0.07605257 -0.0032350985

> summary(get.models(SAFAC1, 1)[[1]])

Call:

lm(formula = FAC1_2 ~ soilNP + tavg_O + tOe + 1, data = tBothFires_plot)

Residuals:

Min 1Q Median 3Q Max

-1.0637 -0.4328 -0.1079 0.3600 2.0374

Coefficients:

Estimate Std. Error t value Pr(>|t|)

(Intercept) -22.06019 5.02362 -4.391 9.48e-05 ***

soilNP -0.17204 0.08536 -2.015 0.0514 .

tavg_O 21.79682 4.09778 5.319 5.65e-06 ***

tOe 6.68360 2.47062 2.705 0.0104 *

---

Signif. codes: 0 ‘***’ 0.001 ‘**’ 0.01 ‘*’ 0.05 ‘.’ 0.1 ‘ ’ 1

Residual standard error: 0.6947 on 36 degrees of freedom

Multiple R-squared: 0.5545, Adjusted R-squared: 0.5174

F-statistic: 14.94 on 3 and 36 DF, p-value: 1.774e-06
