# Supplementary material for: Can Siberian alder N-fixation offset N-loss after severe fire? Quantifying post-fire Siberian alder distribution, growth, and N-fixation in boreal Alaska
Source: PLoS One. 2020 Sep 2;15(9):e0238004. doi: 10.1371/journal.pone.0238004 (PMC7467271; doi:10.1371/journal.pone.0238004)
Supplement: S1 File — (ZIP) [file pone.0238004.s005.zip › AIC_regional_PCA2.docx]

> ## factor 2 model for growth in SA

> factor2.SA = lm(FAC2_2~ soil_BD + soilCN + tsoil_P + tavg_moisture + fire_id , data = tBothFires_plot)

> SAFAC2 <- dredge(factor2.SA, beta = "p", extra = list(

+ "R^2", "*" = function(x) {

+ s <- summary(x)

+ c(Rsq = s$r.squared, adjRsq = s$adj.r.squared,

+ F = s$fstatistic[[1]])

+ })

+ )

Fixed term is "(Intercept)"

> subset(SAFAC2, delta < 2)

Global model call: lm(formula = FAC2_2 ~ soil_BD + soilCN + tsoil_P + tavg_moisture +

fire_id, data = tBothFires_plot)

---

Model selection table

(Int) fir_id sol_BD sCN tvg_mst tsl_P R^2 *.Rsq *.adjRsq *.F df logLik AICc delta weight

14 0 + -0.2478 0.2610 0.4071 0.4071 0.3577 8.240 5 -45.796 103.4 0.00 0.347

10 0 + 0.3271 0.3489 0.3489 0.3137 9.912 4 -47.671 104.5 1.13 0.197

6 0 + -0.3170 0.3425 0.3425 0.3070 9.637 4 -47.865 104.9 1.52 0.163

26 0 + 0.2396 0.1901 0.3831 0.3831 0.3317 7.453 5 -46.589 104.9 1.59 0.157

12 0 + 0.1776 0.3130 0.3788 0.3788 0.3270 7.317 5 -46.730 105.2 1.87 0.136

Models ranked by AICc(x)

> par(mar = c(3,5,6,4))

> plot(SAFAC2, labAsExpr = TRUE)

> summary(model.avg(SAFAC2, subset = delta < 2))

Call:

model.avg(object = SAFAC2, subset = delta < 2)

Component model call:

lm(formula = FAC2_2 ~ <5 unique rhs>, data = tBothFires_plot)

Component models:

df logLik AICc delta weight

134 5 -45.80 103.36 0.00 0.35

14 4 -47.67 104.48 1.13 0.20

13 4 -47.86 104.87 1.52 0.16

145 5 -46.59 104.94 1.59 0.16

124 5 -46.73 105.22 1.87 0.14

Term codes:

fire_id soil_BD soilCN tavg_moisture tsoil_P

1 2 3 4 5

Model-averaged coefficients:

(full average)

Estimate Std. Error Adjusted SE z value Pr(>|z|)

(Intercept) 0.00000 0.00000 0.00000 NA NA

fire_idWDF 0.42840 0.13779 0.14226 3.011 0.0026 **

soilCN -0.13749 0.16649 0.16837 0.817 0.4142

tavg_moisture 0.23536 0.16315 0.16632 1.415 0.1570

tsoil_P 0.02983 0.08726 0.08841 0.337 0.7358

soil_BD 0.02421 0.07869 0.07980 0.303 0.7616

(conditional average)

Estimate Std. Error Adjusted SE z value Pr(>|z|)

(Intercept) 0.0000 0.0000 0.0000 NA NA

fire_idWDF 0.4284 0.1378 0.1423 3.011 0.0026 **

soilCN -0.2699 0.1367 0.1411 1.913 0.0558 .

tavg_moisture 0.2810 0.1376 0.1421 1.977 0.0480 *

tsoil_P 0.1901 0.1344 0.1391 1.367 0.1716

soil_BD 0.1776 0.1349 0.1396 1.273 0.2032

---

Signif. codes: 0 ‘***’ 0.001 ‘**’ 0.01 ‘*’ 0.05 ‘.’ 0.1 ‘ ’ 1

> confint(model.avg(SAFAC2, subset = delta < 2))

2.5 % 97.5 %

(Intercept) 0.000000000 0.000000000

fire_idWDF 0.149583211 0.707223162

soilCN -0.546480408 0.006693591

tavg_moisture 0.002487913 0.559571300

tsoil_P -0.082467730 0.462652128

soil_BD -0.095929180 0.451113886

> model.avg(SAFAC2, subset = cumsum(weight) <= .95)

Call:

model.avg(object = SAFAC2, subset = cumsum(weight) <= 0.95)

Component models:

‘134’ ‘14’ ‘13’ ‘145’ ‘124’ ‘1345’ ‘15’ ‘135’ ‘1234’ ‘1245’ ‘123’ ‘1’ ‘12’ ‘1235’

Coefficients:

(Intercept) fire_idWDF soilCN tavg_moisture tsoil_P soil_BD

full 0 0.432383 -0.1323363 0.1862227 0.06118178 0.02038083

subset 0 0.432383 -0.2394887 0.2729320 0.17757517 0.08627137

> summary(get.models(SAFAC2, 1)[[1]])

Call:

lm(formula = FAC2_2 ~ fire_id + soilCN + tavg_moisture + 1, data = tBothFires_plot)

Residuals:

Min 1Q Median 3Q Max

-1.0786 -0.5810 -0.2367 0.3943 2.1467

Coefficients:

Estimate Std. Error t value Pr(>|t|)

(Intercept) -0.57121 1.20923 -0.472 0.63951

fire_idWDF 0.78886 0.26316 2.998 0.00491 **

soilCN -0.07715 0.04102 -1.881 0.06808 .

tavg_moisture 2.70264 1.36432 1.981 0.05528 .

---

Signif. codes: 0 ‘***’ 0.001 ‘**’ 0.01 ‘*’ 0.05 ‘.’ 0.1 ‘ ’ 1

Residual standard error: 0.8014 on 36 degrees of freedom

Multiple R-squared: 0.4071, Adjusted R-squared: 0.3577

F-statistic: 8.24 on 3 and 36 DF, p-value: 0.0002648
