# Supplementary material for: Can Siberian alder N-fixation offset N-loss after severe fire? Quantifying post-fire Siberian alder distribution, growth, and N-fixation in boreal Alaska
Source: PLoS One. 2020 Sep 2;15(9):e0238004. doi: 10.1371/journal.pone.0238004 (PMC7467271; doi:10.1371/journal.pone.0238004)
Supplement: S1 File — (ZIP) [file pone.0238004.s005.zip › AIC_regional_plant_nfix.docx]

> ## pynifx in study area

> spynfix = lm(PYNFIX ~ tavg_O + tOe + soilNP

+ + fire_id, data = tBothFires_plot)

> sapynfix <- dredge(spynfix, beta = "p", extra = list(

+ "R^2", "*" = function(x) {

+ s <- summary(x)

+ c(Rsq = s$r.squared, adjRsq = s$adj.r.squared,

+ F = s$fstatistic[[1]])

+ })

+ )

Fixed term is "(Intercept)"

> subset(sapynfix, delta < 2)

Global model call: lm(formula = PYNFIX ~ tavg_O + tOe + soilNP + fire_id, data = tBothFires_plot)

---

Model selection table

(Int) fir_id sNP tvg_O tOe R^2 *.Rsq *.adjRsq *.F df logLik AICc delta weight

8 0 + -1.126 1.688 0.4370 0.4370 0.3901 9.314 5 -113.524 238.8 0.00 0.316

6 0 + 2.665 0.3984 0.3984 0.3659 12.250 4 -114.851 238.8 0.03 0.311

16 0 + -1.378 1.985 1.0190 0.4678 0.4678 0.4070 7.691 6 -112.398 239.3 0.53 0.243

14 0 + 2.387 0.6555 0.4115 0.4115 0.3624 8.390 5 -114.411 240.6 1.77 0.130

Models ranked by AICc(x)

> par(mar = c(3,5,6,4))

> plot(sapynfix, labAsExpr = TRUE)

> summary(model.avg(sapynfix, subset = delta < 2))

Call:

model.avg(object = sapynfix, subset = delta < 2)

Component model call:

lm(formula = PYNFIX ~ <4 unique rhs>, data = tBothFires_plot)

Component models:

df logLik AICc delta weight

123 5 -113.52 238.81 0.00 0.32

13 4 -114.85 238.84 0.03 0.31

1234 6 -112.40 239.34 0.53 0.24

134 5 -114.41 240.59 1.77 0.13

Term codes:

fire_id soilNP tavg_O tOe

1 2 3 4

Model-averaged coefficients:

(full average)

Estimate Std. Error Adjusted SE z value Pr(>|z|)

(Intercept) 0.0000 0.0000 0.0000 NA NA

fire_idWDF -2.0210 0.7345 0.7590 2.663 0.00775 **

soilNP -0.6900 0.8195 0.8319 0.829 0.40685

tavg_O 2.1550 0.8257 0.8476 2.542 0.01101 *

tOe 0.3327 0.6258 0.6369 0.522 0.60145

(conditional average)

Estimate Std. Error Adjusted SE z value Pr(>|z|)

(Intercept) 0.0000 0.0000 0.0000 NA NA

fire_idWDF -2.0210 0.7345 0.7590 2.663 0.00775 **

soilNP -1.2353 0.7271 0.7519 1.643 0.10043

tavg_O 2.1550 0.8257 0.8476 2.542 0.01101 *

tOe 0.8923 0.7424 0.7672 1.163 0.24485

---

Signif. codes: 0 ‘***’ 0.001 ‘**’ 0.01 ‘*’ 0.05 ‘.’ 0.1 ‘ ’ 1

> confint(model.avg(sapynfix, subset = delta < 2))

2.5 % 97.5 %

(Intercept) 0.0000000 0.0000000

fire_idWDF -3.5086149 -0.5334834

soilNP -2.7090335 0.2385193

tavg_O 0.4937109 3.8162310

tOe -0.6115082 2.3960495

> model.avg(sapynfix, subset = cumsum(weight) <= .95)

Call:

model.avg(object = sapynfix, subset = cumsum(weight) <= 0.95)

Component models:

‘123’ ‘13’ ‘1234’ ‘134’ ‘12’ ‘3’

Coefficients:

(Intercept) fire_idWDF soilNP tavg_O tOe

full 0 -1.996437 -0.7673707 2.053328 0.3013860

subset 0 -2.066062 -1.3548162 2.185112 0.8922706

> summary(get.models(sapynfix, 1)[[1]])

Call:

lm(formula = PYNFIX ~ fire_id + soilNP + tavg_O + 1, data = tBothFires_plot)

Residuals:

Min 1Q Median 3Q Max

-5.1040 -3.5280 -0.8561 2.5960 14.7863

Coefficients:

Estimate Std. Error t value Pr(>|t|)

(Intercept) -25.6892 16.3691 -1.569 0.12531

fire_idWDF -4.4031 1.4672 -3.001 0.00486 **

soilNP -0.8418 0.5357 -1.571 0.12488

tavg_O 51.0779 21.6773 2.356 0.02402 *

---

Signif. codes: 0 ‘***’ 0.001 ‘**’ 0.01 ‘*’ 0.05 ‘.’ 0.1 ‘ ’ 1

Residual standard error: 4.357 on 36 degrees of freedom

Multiple R-squared: 0.437, Adjusted R-squared: 0.3901

F-statistic: 9.314 on 3 and 36 DF, p-value: 0.0001078
