# Supplementary material for: Can Siberian alder N-fixation offset N-loss after severe fire? Quantifying post-fire Siberian alder distribution, growth, and N-fixation in boreal Alaska
Source: PLoS One. 2020 Sep 2;15(9):e0238004. doi: 10.1371/journal.pone.0238004 (PMC7467271; doi:10.1371/journal.pone.0238004)
Supplement: S1 File — (ZIP) [file pone.0238004.s005.zip › AIC_WDF_density.docx]

> ## min_dist model in wickersham dome fire

> wmin_dist = lm(min_dist~slope + tavg_moisture

+ , data = tWDF_plot)

> wdfmin_dist <- dredge(wmin_dist, beta = "p", extra = list(

+ "R^2", "*" = function(x) {

+ s <- summary(x)

+ c(Rsq = s$r.squared, adjRsq = s$adj.r.squared,

+ F = s$fstatistic[[1]])

+ })

+ )

Fixed term is "(Intercept)"

> subset(wdfmin_dist, delta < 2)

Global model call: lm(formula = min_dist ~ slope + tavg_moisture, data = tWDF_plot)

---

Model selection table

(Int) slp tvg_mst R^2 *.Rsq *.adjRsq *.F df logLik AICc delta weight

4 0 -7.303 -9.907 0.4476 0.4476 0.3862 7.292 4 -83.603 177.7 0 1

Models ranked by AICc(x)

> par(mar = c(3,5,6,4))

> plot(wdfmin_dist, labAsExpr = TRUE)

> summary(model.avg(wdfmin_dist, subset = delta < 2))

Error in model.avg.model.selection(wdfmin_dist, subset = delta < 2) :

'object' consists of only one model

> summary(model.avg(wdfmin_dist, subset = cumsum(weight) <= .95))

Call:

model.avg(object = wdfmin_dist, subset = cumsum(weight) <= 0.95)

Component model call:

lm(formula = min_dist ~ <2 unique rhs>, data = tWDF_plot)

Component models:

df logLik AICc delta weight

12 4 -83.60 177.71 0.00 0.75

2 3 -86.25 179.92 2.21 0.25

Term codes:

slope tavg_moisture

1 2

Model-averaged coefficients:

(full average)

Estimate Std. Error Adjusted SE z value Pr(>|z|)

(Intercept) 0.000 0.000 0.000 NA NA

slope -5.488 4.209 4.344 1.263 0.20649

tavg_moisture -9.832 3.278 3.509 2.802 0.00508 **

(conditional average)

Estimate Std. Error Adjusted SE z value Pr(>|z|)

(Intercept) 0.000 0.000 0.000 NA NA

slope -7.303 3.212 3.443 2.121 0.03393 *

tavg_moisture -9.832 3.278 3.509 2.802 0.00508 **

---

Signif. codes: 0 ‘***’ 0.001 ‘**’ 0.01 ‘*’ 0.05 ‘.’ 0.1 ‘ ’ 1

> summary(get.models(wdfmin_dist, 1)[[1]])

Call:

lm(formula = min_dist ~ slope + tavg_moisture + 1, data = tWDF_plot)

Residuals:

Min 1Q Median 3Q Max

-16.8462 -12.4811 0.1018 9.2498 26.5615

Coefficients:

Estimate Std. Error t value Pr(>|t|)

(Intercept) 79.5664 18.3036 4.347 0.000388 ***

slope -1.4853 0.6533 -2.273 0.035482 *

tavg_moisture -91.8818 29.7944 -3.084 0.006401 **

---

Signif. codes: 0 ‘***’ 0.001 ‘**’ 0.01 ‘*’ 0.05 ‘.’ 0.1 ‘ ’ 1

Residual standard error: 14 on 18 degrees of freedom

Multiple R-squared: 0.4476, Adjusted R-squared: 0.3862

F-statistic: 7.292 on 2 and 18 DF, p-value: 0.004791
