# Supplementary material for: Can Siberian alder N-fixation offset N-loss after severe fire? Quantifying post-fire Siberian alder distribution, growth, and N-fixation in boreal Alaska
Source: PLoS One. 2020 Sep 2;15(9):e0238004. doi: 10.1371/journal.pone.0238004 (PMC7467271; doi:10.1371/journal.pone.0238004)
Supplement: S1 File — (ZIP) [file pone.0238004.s005.zip › AIC_WDF_nodule_nfix.docx]

> ## N-fix in WDF

> wnfix = lm(NFIX~ soilNP + elev , data = tWDF_plot)

> wdfnfix <- dredge(wnfix, beta = "p", extra = list(

+ "R^2", "*" = function(x) {

+ s <- summary(x)

+ c(Rsq = s$r.squared, adjRsq = s$adj.r.squared,

+ F = s$fstatistic[[1]])

+ })

+ )

Fixed term is "(Intercept)"

> subset(wdfnfix, delta < 2)

Global model call: lm(formula = NFIX ~ soilNP + elev, data = tWDF_plot)

---

Model selection table

(Intrc) elev R^2 *.Rsq *.adjRsq *.F df logLik AICc delta weight

2 0 1.898 0.4934 0.4934 0.4667 18.5 3 -43.015 93.4 0 1

Models ranked by AICc(x)

> par(mar = c(3,5,6,4))

> plot(wdfnfix, labAsExpr = TRUE)

> summary(model.avg(wdfnfix, subset = delta < 2))

Error in model.avg.model.selection(wdfnfix, subset = delta < 2) :

'object' consists of only one model

> model.avg(wdfnfix, subset = cumsum(weight) <= .95)

Error in model.avg.model.selection(wdfnfix, subset = cumsum(weight) <= :

'object' consists of only one model

> summary(get.models(wdfnfix, 1)[[1]])

Call:

lm(formula = NFIX ~ elev + 1, data = tWDF_plot)

Residuals:

Min 1Q Median 3Q Max

-4.8565 -0.6834 -0.2278 0.6245 4.4629

Coefficients:

Estimate Std. Error t value Pr(>|t|)

(Intercept) -4.338330 2.804161 -1.547 0.138333

elev 0.027859 0.006477 4.301 0.000385 ***

---

Signif. codes: 0 ‘***’ 0.001 ‘**’ 0.01 ‘*’ 0.05 ‘.’ 0.1 ‘ ’ 1

Residual standard error: 1.973 on 19 degrees of freedom

Multiple R-squared: 0.4934, Adjusted R-squared: 0.4667

F-statistic: 18.5 on 1 and 19 DF, p-value: 0.0003851
