# Supplementary material for: Can Siberian alder N-fixation offset N-loss after severe fire? Quantifying post-fire Siberian alder distribution, growth, and N-fixation in boreal Alaska
Source: PLoS One. 2020 Sep 2;15(9):e0238004. doi: 10.1371/journal.pone.0238004 (PMC7467271; doi:10.1371/journal.pone.0238004)
Supplement: S1 File — (ZIP) [file pone.0238004.s005.zip › AIC_WDF_PCA1.docx]

> ## factor 1 model for growth in WDF

> factor1.WDF = lm(FAC1_2~ tavg_O , data = tWDF_plot)

> WDFFAC1 <- dredge(factor1.WDF, beta = "p", extra = list(

+ "R^2", "*" = function(x) {

+ s <- summary(x)

+ c(Rsq = s$r.squared, adjRsq = s$adj.r.squared,

+ F = s$fstatistic[[1]])

+ })

+ )

Fixed term is "(Intercept)"

> subset(WDFFAC1, delta < 2)

Global model call: lm(formula = FAC1_2 ~ tavg_O, data = tWDF_plot)

---

Model selection table

(Int) tvg_O R^2 *.Rsq *.adjRsq *.F df logLik AICc delta weight

2 0 0.5849 0.3215 0.3215 0.2858 9.005 3 -25.862 59.1 0 1

Models ranked by AICc(x)

> par(mar = c(3,5,6,4))

> plot(WDFFAC1, labAsExpr = TRUE)

> summary(model.avg(WDFFAC1, subset = delta < 2))

Error in model.avg.model.selection(WDFFAC1, subset = delta < 2) :

'object' consists of only one model

> model.avg(WDFFAC1, subset = cumsum(weight) <= .95)

Error in model.avg.model.selection(WDFFAC1, subset = cumsum(weight) <= :

'object' consists of only one model

> summary(get.models(WDFFAC1, 1)[[1]])

Call:

lm(formula = FAC1_2 ~ tavg_O + 1, data = tWDF_plot)

Residuals:

Min 1Q Median 3Q Max

-1.4182 -0.5997 0.0239 0.3463 1.9910

Coefficients:

Estimate Std. Error t value Pr(>|t|)

(Intercept) -9.898 3.672 -2.695 0.01434 *

tavg_O 16.031 5.342 3.001 0.00735 **

---

Signif. codes: 0 ‘***’ 0.001 ‘**’ 0.01 ‘*’ 0.05 ‘.’ 0.1 ‘ ’ 1

Residual standard error: 0.8717 on 19 degrees of freedom

Multiple R-squared: 0.3215, Adjusted R-squared: 0.2858

F-statistic: 9.005 on 1 and 19 DF, p-value: 0.007349
