# Supplementary material for: Can Siberian alder N-fixation offset N-loss after severe fire? Quantifying post-fire Siberian alder distribution, growth, and N-fixation in boreal Alaska
Source: PLoS One. 2020 Sep 2;15(9):e0238004. doi: 10.1371/journal.pone.0238004 (PMC7467271; doi:10.1371/journal.pone.0238004)
Supplement: S1 File — (ZIP) [file pone.0238004.s005.zip › AIC_WDF_PCA2.docx]

> ## factor 2 model for growth in WDF

> factor2.WDF = lm(FAC2_2~ tavg_moisture + tsoil_P + soilCN + Oa, data = tWDF_plot)

> WDFFAC2 <- dredge(factor2.WDF, beta = "p", extra = list(

+ "R^2", "*" = function(x) {

+ s <- summary(x)

+ c(Rsq = s$r.squared, adjRsq = s$adj.r.squared,

+ F = s$fstatistic[[1]])

+ })

+ )

Fixed term is "(Intercept)"

> subset(WDFFAC2, delta < 2)

Global model call: lm(formula = FAC2_2 ~ tavg_moisture + tsoil_P + soilCN + Oa,

data = tWDF_plot)

---

Model selection table

(Int) sCN tvg_mst R^2 *.Rsq *.adjRsq *.F df logLik AICc delta weight

3 0 -0.6459 0.3425 0.3425 0.3078 9.895 3 -26.956 61.3 0 0.5

7 0 -0.5128 0.3396 0.4324 0.4324 0.3693 6.856 4 -25.412 61.3 0 0.5

Models ranked by AICc(x)

> par(mar = c(3,5,6,4))

> plot(WDFFAC2, labAsExpr = TRUE)

> summary(model.avg(WDFFAC2, subset = delta < 2))

Call:

model.avg(object = WDFFAC2, subset = delta < 2)

Component model call:

lm(formula = FAC2_2 ~ <2 unique rhs>, data = tWDF_plot)

Component models:

df logLik AICc delta weight

1 3 -26.96 61.32 0 0.5

12 4 -25.41 61.32 0 0.5

Term codes:

soilCN tavg_moisture

1 2

Model-averaged coefficients:

(full average)

Estimate Std. Error Adjusted SE z value Pr(>|z|)

(Intercept) 0.0000 0.0000 0.0000 NA NA

soilCN -0.5793 0.2138 0.2274 2.548 0.0108 *

tavg_moisture 0.1698 0.2215 0.2282 0.744 0.4568

(conditional average)

Estimate Std. Error Adjusted SE z value Pr(>|z|)

(Intercept) 0.0000 0.0000 0.0000 NA NA

soilCN -0.5793 0.2138 0.2274 2.548 0.0108 *

tavg_moisture 0.3396 0.2011 0.2156 1.575 0.1152

---

Signif. codes: 0 ‘***’ 0.001 ‘**’ 0.01 ‘*’ 0.05 ‘.’ 0.1 ‘ ’ 1

> confint(model.avg(WDFFAC2, subset = delta < 2))

2.5 % 97.5 %

(Intercept) 0.00000000 0.0000000

soilCN -1.02498296 -0.1337035

tavg_moisture -0.08291209 0.7620302

> model.avg(WDFFAC2, subset = cumsum(weight) <= .95)

Call:

model.avg(object = WDFFAC2, subset = cumsum(weight) <= 0.95)

Component models:

‘2’ ‘23’ ‘24’ ‘12’ ‘123’ ‘13’ ‘234’ ‘3’ ‘4’ ‘14’ ‘34’ ‘124’

Coefficients:

(Intercept) soilCN tavg_moisture tsoil_P Oa

full 0 -0.4298620 0.1829862 0.06230276 -0.06373439

subset 0 -0.5293604 0.3695620 0.25317379 -0.24524821

> summary(get.models(WDFFAC2, 1)[[1]])

Call:

lm(formula = FAC2_2 ~ soilCN + 1, data = tWDF_plot)

Residuals:

Min 1Q Median 3Q Max

-1.2248 -0.6845 -0.2157 0.4923 2.2096

Coefficients:

Estimate Std. Error t value Pr(>|t|)

(Intercept) 4.71212 1.10476 4.265 0.000418 ***

soilCN -0.20279 0.06447 -3.146 0.005323 **

---

Signif. codes: 0 ‘***’ 0.001 ‘**’ 0.01 ‘*’ 0.05 ‘.’ 0.1 ‘ ’ 1

Residual standard error: 0.9182 on 19 degrees of freedom

Multiple R-squared: 0.3425, Adjusted R-squared: 0.3078

F-statistic: 9.895 on 1 and 19 DF, p-value: 0.005323
