# Supplementary material for: Can Siberian alder N-fixation offset N-loss after severe fire? Quantifying post-fire Siberian alder distribution, growth, and N-fixation in boreal Alaska
Source: PLoS One. 2020 Sep 2;15(9):e0238004. doi: 10.1371/journal.pone.0238004 (PMC7467271; doi:10.1371/journal.pone.0238004)
Supplement: S1 File — (ZIP) [file pone.0238004.s005.zip › AIC_WDF_plant_nfix.docx]

> ## pynifx in WDF

> Wpynfix = lm(PYNFIX ~ tavg_O + elev, data = tWDF_plot)

> wdfpynfix <- dredge(Wpynfix, beta = "p", extra = list(

+ "R^2", "*" = function(x) {

+ s <- summary(x)

+ c(Rsq = s$r.squared, adjRsq = s$adj.r.squared,

+ F = s$fstatistic[[1]])

+ })

+ )

Fixed term is "(Intercept)"

> subset(wdfpynfix, delta < 2)

Global model call: lm(formula = PYNFIX ~ tavg_O + elev, data = tWDF_plot)

---

Model selection table

(Int) elv tvg_O R^2 *.Rsq *.adjRsq *.F df logLik AICc delta weight

2 0 0.8671 0.2078 0.2078 0.1661 4.983 3 -40.344 88.1 0.00 0.464

3 0 0.8024 0.1779 0.1779 0.1347 4.113 3 -40.732 88.9 0.78 0.315

4 0 0.5797 0.4717 0.2662 0.2662 0.1846 3.265 4 -39.540 89.6 1.48 0.221

Models ranked by AICc(x)

> par(mar = c(3,5,6,4))

> plot(wdfpynfix, labAsExpr = TRUE)

> summary(model.avg(wdfpynfix, subset = delta < 2))

Call:

model.avg(object = wdfpynfix, subset = delta < 2)

Component model call:

lm(formula = PYNFIX ~ <3 unique rhs>, data = tWDF_plot)

Component models:

df logLik AICc delta weight

1 3 -40.34 88.10 0.00 0.46

2 3 -40.73 88.88 0.78 0.31

12 4 -39.54 89.58 1.48 0.22

Term codes:

elev tavg_O

1 2

Model-averaged coefficients:

(full average)

Estimate Std. Error Adjusted SE z value Pr(>|z|)

(Intercept) 0.0000 0.0000 0.0000 NA NA

elev 0.5306 0.4960 0.5109 1.039 0.299

tavg_O 0.3570 0.4562 0.4692 0.761 0.447

(conditional average)

Estimate Std. Error Adjusted SE z value Pr(>|z|)

(Intercept) 0.0000 0.0000 0.0000 NA NA

elev 0.7742 0.4127 0.4384 1.766 0.0774 .

tavg_O 0.6659 0.4273 0.4528 1.471 0.1414

---

Signif. codes: 0 ‘***’ 0.001 ‘**’ 0.01 ‘*’ 0.05 ‘.’ 0.1 ‘ ’ 1

> confint(model.avg(wdfpynfix, subset = delta < 2))

2.5 % 97.5 %

(Intercept) 0.00000000 0.000000

elev -0.08495149 1.633439

tavg_O -0.22155957 1.553286

> model.avg(wdfpynfix, subset = cumsum(weight) <= .95)

Call:

model.avg(object = wdfpynfix, subset = cumsum(weight) <= 0.95)

Component models:

‘1’ ‘2’ ‘12’

Coefficients:

(Intercept) elev tavg_O

full 0 0.5305677 0.3569666

subset 0 0.7742435 0.6658634

> summary(get.models(wdfpynfix, 1)[[1]])

Call:

lm(formula = PYNFIX ~ elev + 1, data = tWDF_plot)

Residuals:

Min 1Q Median 3Q Max

-2.4669 -1.0055 -0.2857 0.9469 4.0949

Coefficients:

Estimate Std. Error t value Pr(>|t|)

(Intercept) -3.214574 2.469172 -1.302 0.2085

elev 0.012730 0.005703 2.232 0.0378 *

---

Signif. codes: 0 ‘***’ 0.001 ‘**’ 0.01 ‘*’ 0.05 ‘.’ 0.1 ‘ ’ 1

Residual standard error: 1.737 on 19 degrees of freedom

Multiple R-squared: 0.2078, Adjusted R-squared: 0.1661

F-statistic: 4.983 on 1 and 19 DF, p-value: 0.03784
