# Supplementary material for: Can Siberian alder N-fixation offset N-loss after severe fire? Quantifying post-fire Siberian alder distribution, growth, and N-fixation in boreal Alaska
Source: PLoS One. 2020 Sep 2;15(9):e0238004. doi: 10.1371/journal.pone.0238004 (PMC7467271; doi:10.1371/journal.pone.0238004)
Supplement: S2 File — (ZIP) [file pone.0238004.s006.zip › SEM_BF_density.docx]

# SEM output for alder plant density in Boundary Fire black spruce plots:

> library(lavaan)

This is lavaan 0.5-22

lavaan is BETA software! Please report any bugs.

Warning message:

package ‘lavaan’ was built under R version 3.3.2

> ## just black spruce BF plot density SEM

> tBF_plot_bs <- read.csv('I:/Thesis/Manuscript1/PLOS_ONE/regression_results/SEM/tBF_plot_bs.csv')

> bfdensity_bs <- '

+ min_dist ~ tOe

+ tOe ~ zonal_dNBR +tavg_O

+ tavg_O ~ zonal_dNBR

+ '

> bfdensity_bs.fit <- sem(bfdensity_bs, data = tBF_plot_bs)

Warning message:

In lav_data_full(data = data, group = group, group.label = group.label, :

lavaan WARNING: some observed variances are (at least) a factor 1000 times larger than others; use varTable(fit) to investigate

> summary (bfdensity_bs.fit, standardized = TRUE)

lavaan (0.5-22) converged normally after 68 iterations

Number of observations 11

Estimator ML

Minimum Function Test Statistic 3.468

Degrees of freedom 2

P-value (Chi-square) 0.177

Parameter Estimates:

Information Expected

Standard Errors Standard

Regressions:

Estimate Std.Err z-value P(>|z|) Std.lv Std.all

min_dist ~

tOe -121.755 42.476 -2.866 0.004 -121.755 -0.654

tOe ~

zonal_dNBR -0.000 0.000 -1.765 0.078 -0.000 -0.345

tavg_O -1.641 0.533 -3.078 0.002 -1.641 -0.601

tavg_O ~

zonal_dNBR 0.000 0.000 2.222 0.026 0.000 0.557

Variances:

Estimate Std.Err z-value P(>|z|) Std.lv Std.all

.min_dist 130.594 55.686 2.345 0.019 130.594 0.572

.tOe 0.002 0.001 2.345 0.019 0.002 0.289

.tavg_O 0.001 0.000 2.345 0.019 0.001 0.690

> summary (bfdensity_bs.fit, modindices = TRUE)

lavaan (0.5-22) converged normally after 68 iterations

Number of observations 11

Estimator ML

Minimum Function Test Statistic 3.468

Degrees of freedom 2

P-value (Chi-square) 0.177

Parameter Estimates:

Information Expected

Standard Errors Standard

Regressions:

Estimate Std.Err z-value P(>|z|)

min_dist ~

tOe -121.755 42.476 -2.866 0.004

tOe ~

zonal_dNBR -0.000 0.000 -1.765 0.078

tavg_O -1.641 0.533 -3.078 0.002

tavg_O ~

zonal_dNBR 0.000 0.000 2.222 0.026

Variances:

Estimate Std.Err z-value P(>|z|)

.min_dist 130.594 55.686 2.345 0.019

.tOe 0.002 0.001 2.345 0.019

.tavg_O 0.001 0.000 2.345 0.019

Modification Indices:

lhs op rhs mi epc sepc.lv sepc.all sepc.nox

9 min_dist ~~ tOe 0.087 0.052 0.052 0.043 0.043

10 min_dist ~~ tavg_O 2.167 -0.144 -0.144 -0.322 -0.322

12 min_dist ~ tavg_O 0.494 -133.774 -133.774 -0.263 -0.263

13 min_dist ~ zonal_dNBR 2.387 0.062 0.062 0.480 0.004

14 tOe ~ min_dist 0.087 0.000 0.000 0.075 0.075

15 tavg_O ~ min_dist 2.167 -0.001 -0.001 -0.562 -0.562

17 zonal_dNBR ~ min_dist 1.048 2.848 2.848 0.368 0.368

18 zonal_dNBR ~ tOe 0.000 0.000 0.000 0.000 0.000

19 zonal_dNBR ~ tavg_O 0.000 0.001 0.001 0.000 0.000

> inspect(bfdensity_bs.fit ,'r2')

min_dist tOe tavg_O

0.428 0.711 0.310
