# Supplementary material for: Can Siberian alder N-fixation offset N-loss after severe fire? Quantifying post-fire Siberian alder distribution, growth, and N-fixation in boreal Alaska
Source: PLoS One. 2020 Sep 2;15(9):e0238004. doi: 10.1371/journal.pone.0238004 (PMC7467271; doi:10.1371/journal.pone.0238004)
Supplement: S2 File — (ZIP) [file pone.0238004.s006.zip › SEM_BF_PCA1.docx]

# SEM output for PCA1 in Boundary Fire black spruce plots:

> ## BF plot PCA1 SEM in black spruce

> bfpca1_bs <- '

+ FAC1_2 ~ soil_pH + tavg_O + avg_moisture + zonal_dNBR

+ tavg_O ~ zonal_dNBR

+ '

> bfpca1_bs.fit <- sem(bfpca1_bs, data = tBF_plot_bs)

> summary (bfpca1_bs.fit, standardized = TRUE)

lavaan (0.5-22) converged normally after 44 iterations

Number of observations 11

Estimator ML

Minimum Function Test Statistic 3.623

Degrees of freedom 2

P-value (Chi-square) 0.163

Parameter Estimates:

Information Expected

Standard Errors Standard

Regressions:

Estimate Std.Err z-value P(>|z|) Std.lv Std.all

FAC1_2 ~

soil_pH -0.515 0.154 -3.347 0.001 -0.515 -0.274

tavg_O 9.695 1.635 5.930 0.000 9.695 0.532

avg_moisture -0.151 0.014 -11.044 0.000 -0.151 -0.835

zonal_dNBR -0.002 0.000 -4.580 0.000 -0.002 -0.443

tavg_O ~

zonal_dNBR 0.000 0.000 2.222 0.026 0.000 0.557

Variances:

Estimate Std.Err z-value P(>|z|) Std.lv Std.all

.FAC1_2 0.018 0.008 2.345 0.019 0.018 0.061

.tavg_O 0.001 0.000 2.345 0.019 0.001 0.690

> summary (bfpca1_bs.fit, modindices = TRUE)

lavaan (0.5-22) converged normally after 44 iterations

Number of observations 11

Estimator ML

Minimum Function Test Statistic 3.623

Degrees of freedom 2

P-value (Chi-square) 0.163

Parameter Estimates:

Information Expected

Standard Errors Standard

Regressions:

Estimate Std.Err z-value P(>|z|)

FAC1_2 ~

soil_pH -0.515 0.154 -3.347 0.001

tavg_O 9.695 1.635 5.930 0.000

avg_moisture -0.151 0.014 -11.044 0.000

zonal_dNBR -0.002 0.000 -4.580 0.000

tavg_O ~

zonal_dNBR 0.000 0.000 2.222 0.026

Variances:

Estimate Std.Err z-value P(>|z|)

.FAC1_2 0.018 0.008 2.345 0.019

.tavg_O 0.001 0.000 2.345 0.019

Modification Indices:

lhs op rhs mi epc sepc.lv sepc.all sepc.nox

8 soil_pH ~~ soil_pH 0.000 0.000 0.000 0.000 0.000

9 soil_pH ~~ avg_moisture 0.000 0.000 0.000 0.000 0.000

10 soil_pH ~~ zonal_dNBR 0.000 0.000 0.000 0.000 0.000

11 avg_moisture ~~ avg_moisture 0.000 0.000 0.000 0.000 0.000

12 avg_moisture ~~ zonal_dNBR 0.000 0.000 0.000 0.000 0.000

15 tavg_O ~ FAC1_2 1.112 0.017 0.017 0.309 0.309

16 tavg_O ~ soil_pH 2.691 -0.047 -0.047 -0.451 -1.566

17 tavg_O ~ avg_moisture 0.359 -0.002 -0.002 -0.152 -0.051

18 soil_pH ~ FAC1_2 0.532 -0.107 -0.107 -0.200 -0.200

19 soil_pH ~ tavg_O 1.883 -3.655 -3.655 -0.377 -0.377

20 soil_pH ~ avg_moisture 0.000 0.000 0.000 0.000 0.000

21 soil_pH ~ zonal_dNBR 0.000 0.000 0.000 0.000 0.000

22 avg_moisture ~ FAC1_2 0.048 -0.283 -0.283 -0.051 -0.051

23 avg_moisture ~ tavg_O 0.273 -15.659 -15.659 -0.155 -0.155

24 avg_moisture ~ soil_pH 0.000 0.000 0.000 0.000 0.000

25 avg_moisture ~ zonal_dNBR 0.000 0.000 0.000 0.000 0.000

26 zonal_dNBR ~ FAC1_2 0.144 -24.877 -24.877 -0.115 -0.115

27 zonal_dNBR ~ tavg_O 0.587 -982.961 -982.961 -0.250 -0.250

28 zonal_dNBR ~ soil_pH 0.000 0.000 0.000 0.000 0.000

29 zonal_dNBR ~ avg_moisture 0.000 0.000 0.000 0.000 0.000

> inspect(bfpca1_bs.fit ,'r2')

FAC1_2 tavg_O

0.939 0.310
