# Supplementary material for: Can Siberian alder N-fixation offset N-loss after severe fire? Quantifying post-fire Siberian alder distribution, growth, and N-fixation in boreal Alaska
Source: PLoS One. 2020 Sep 2;15(9):e0238004. doi: 10.1371/journal.pone.0238004 (PMC7467271; doi:10.1371/journal.pone.0238004)
Supplement: S2 File — (ZIP) [file pone.0238004.s006.zip › SEM_BF_plant_nfix.docx]

# SEM output for plant-level N-fixation in Boundary Fire black spruce plots:

> ## black spruce plots only for pynfix SEM model

> bfpynfix_bs <- '

+ PYNFIX ~ lnbio + NFIX

+ PYNFIX ~ tavg_O

+ lnbio ~ tavg_O

+ tavg_O ~ zonal_dNBR + soilNP

+ NFIX ~ soilCN

+ '

> bfpynfix_bs.fit <- sem(bfpynfix_bs, data = tBF_plot_bs)

Warning message:

In lav_data_full(data = data, group = group, group.label = group.label, :

lavaan WARNING: some observed variances are (at least) a factor 1000 times larger than others; use varTable(fit) to investigate

> summary (bfpynfix_bs.fit, standardized = TRUE)

lavaan (0.5-22) converged normally after 77 iterations

Number of observations 11

Estimator ML

Minimum Function Test Statistic 12.552

Degrees of freedom 11

P-value (Chi-square) 0.324

Parameter Estimates:

Information Expected

Standard Errors Standard

Regressions:

Estimate Std.Err z-value P(>|z|) Std.lv Std.all

PYNFIX ~

lnbio 0.330 0.018 18.483 0.000 0.330 0.798

NFIX 0.348 0.031 11.084 0.000 0.348 0.374

tavg_O 24.240 7.934 3.055 0.002 24.240 0.133

lnbio ~

tavg_O 280.089 103.425 2.708 0.007 280.089 0.632

tavg_O ~

zonal_dNBR 0.000 0.000 3.529 0.000 0.000 0.659

soilNP -0.012 0.004 -3.075 0.002 -0.012 -0.574

NFIX ~

soilCN -0.828 0.422 -1.963 0.050 -0.828 -0.509

Variances:

Estimate Std.Err z-value P(>|z|) Std.lv Std.all

.PYNFIX 0.363 0.155 2.345 0.019 0.363 0.012

.lnbio 103.835 44.276 2.345 0.019 103.835 0.600

.tavg_O 0.000 0.000 2.345 0.019 0.000 0.371

.NFIX 25.145 10.722 2.345 0.019 25.145 0.741

> summary (bfpynfix_bs.fit, modindices = TRUE)

lavaan (0.5-22) converged normally after 77 iterations

Number of observations 11

Estimator ML

Minimum Function Test Statistic 12.552

Degrees of freedom 11

P-value (Chi-square) 0.324

Parameter Estimates:

Information Expected

Standard Errors Standard

Regressions:

Estimate Std.Err z-value P(>|z|)

PYNFIX ~

lnbio 0.330 0.018 18.483 0.000

NFIX 0.348 0.031 11.084 0.000

tavg_O 24.240 7.934 3.055 0.002

lnbio ~

tavg_O 280.089 103.425 2.708 0.007

tavg_O ~

zonal_dNBR 0.000 0.000 3.529 0.000

soilNP -0.012 0.004 -3.075 0.002

NFIX ~

soilCN -0.828 0.422 -1.963 0.050

Variances:

Estimate Std.Err z-value P(>|z|)

.PYNFIX 0.363 0.155 2.345 0.019

.lnbio 103.835 44.276 2.345 0.019

.tavg_O 0.000 0.000 2.345 0.019

.NFIX 25.145 10.722 2.345 0.019

Modification Indices:

lhs op rhs mi epc sepc.lv sepc.all sepc.nox

13 zonal_dNBR ~~ soilNP 0.000 0.000 0.000 0.000 0.000

14 zonal_dNBR ~~ soilCN 0.000 0.000 0.000 0.000 0.000

15 soilNP ~~ soilNP 0.000 0.000 0.000 0.000 0.000

16 soilNP ~~ soilCN 0.000 0.000 0.000 0.000 0.000

17 soilCN ~~ soilCN 0.000 0.000 0.000 0.000 0.000

19 PYNFIX ~~ tavg_O 0.011 0.000 0.000 -0.003 -0.003

20 PYNFIX ~~ NFIX 1.958 2.563 2.563 0.081 0.081

21 lnbio ~~ tavg_O 0.249 0.035 0.035 0.090 0.090

22 lnbio ~~ NFIX 0.430 -10.099 -10.099 -0.132 -0.132

23 tavg_O ~~ NFIX 2.139 0.040 0.040 0.231 0.231

24 PYNFIX ~ zonal_dNBR 0.866 0.002 0.002 0.039 0.000

25 PYNFIX ~ soilNP 0.879 0.151 0.151 0.039 0.028

26 PYNFIX ~ soilCN 1.958 0.084 0.084 0.056 0.016

27 lnbio ~ PYNFIX 0.124 -0.538 -0.538 -0.222 -0.222

28 lnbio ~ NFIX 0.124 -0.187 -0.187 -0.083 -0.083

29 lnbio ~ zonal_dNBR 1.765 -0.042 -0.042 -0.373 -0.003

30 lnbio ~ soilNP 0.905 -2.363 -2.363 -0.250 -0.180

31 lnbio ~ soilCN 0.188 -0.384 -0.384 -0.105 -0.029

32 tavg_O ~ PYNFIX 0.911 0.002 0.002 0.292 0.292

33 tavg_O ~ lnbio 0.249 0.000 0.000 0.149 0.149

34 tavg_O ~ NFIX 1.009 0.001 0.001 0.205 0.205

35 tavg_O ~ soilCN 1.756 0.004 0.004 0.465 0.130

36 NFIX ~ PYNFIX 0.402 0.202 0.202 0.188 0.188

37 NFIX ~ lnbio 0.139 0.043 0.043 0.098 0.098

38 NFIX ~ tavg_O 2.045 75.080 75.080 0.383 0.383

39 NFIX ~ zonal_dNBR 0.069 0.004 0.004 0.072 0.001

40 NFIX ~ soilNP 1.067 -1.998 -1.998 -0.477 -0.343

41 zonal_dNBR ~ PYNFIX 0.931 -6.146 -6.146 -0.286 -0.286

42 zonal_dNBR ~ lnbio 1.627 -3.509 -3.509 -0.395 -0.395

43 zonal_dNBR ~ tavg_O 0.082 -305.237 -305.237 -0.078 -0.078

44 zonal_dNBR ~ NFIX 0.002 0.230 0.230 0.011 0.011

45 zonal_dNBR ~ soilNP 0.000 0.000 0.000 0.000 0.000

46 zonal_dNBR ~ soilCN 0.000 0.000 0.000 0.000 0.000

47 soilNP ~ PYNFIX 2.289 -0.066 -0.066 -0.257 -0.257

48 soilNP ~ lnbio 1.793 -0.024 -0.024 -0.230 -0.230

49 soilNP ~ tavg_O 0.456 -5.282 -5.282 -0.113 -0.113

50 soilNP ~ NFIX 0.741 -0.034 -0.034 -0.143 -0.143

51 soilNP ~ zonal_dNBR 0.000 0.000 0.000 0.000 0.000

52 soilNP ~ soilCN 0.000 0.000 0.000 0.000 0.000

53 soilCN ~ PYNFIX 2.048 0.151 0.151 0.228 0.228

54 soilCN ~ lnbio 1.477 0.052 0.052 0.192 0.192

55 soilCN ~ tavg_O 0.652 15.385 15.385 0.128 0.128

56 soilCN ~ NFIX 0.517 0.075 0.075 0.122 0.122

57 soilCN ~ zonal_dNBR 0.000 0.000 0.000 0.000 0.000

58 soilCN ~ soilNP 0.000 0.000 0.000 0.000 0.000

> inspect( bfpynfix_bs.fit,'r2')

PYNFIX lnbio tavg_O NFIX

0.988 0.400 0.629 0.259
